# Unraveling the Effect of Soil Moisture on Microbial Diversity and Enzymatic Activity in Agricultural Soils

**DOI:** 10.3390/microorganisms13061245

**Published:** 2025-05-28

**Authors:** Kalisa Amarsingh Bogati, Piotr Sewerniak, Maciej Walczak

**Affiliations:** 1 Department of Environmental Microbiology and Biotechnology, Faculty of Biological and Veterinary Sciences, Nicolaus Copernicus University, 87-100 Toruń, Poland; 2 Department of Biology and Biotechnology, Worcester Polytechnic Institute, Life Sciences and Bioengineering Center, 60 Prescott Street, Worcester, MA 01605, USA; 3 Department Cell Biology and Biochemistry, Uniwersytet Kazimierza Wielkiego, Wydział Nauk o Polityce i Administracji ul. ks. J. Poniatowskiego 12, 85-671 Bydgoszcz, Poland; 4 Department of Soil Science and Landscape Management, Faculty of Earth Science and Spatial Management, Nicolaus Copernicus University, 87-100 Toruń, Poland; sewern@umk.pl; 5 Bacto-Tech Sp. z o.o., Polna 148, 87-100 Toruń, Poland

**Keywords:** soil science, cropland, drought, soil microbes, microbial enzymes, functional diversity

## Abstract

This study investigates the impact of two months of drought stress on the microbial diversity, enzyme activities and functional diversity in four agricultural soils (Gniewkowo (G); Lulkowo (L); Nieszawa (N); Suchatówka (S)) from Poland during summer season. The physicochemical parameters (pH, organic carbon, calcium carbonate, total nitrogen, nitrate, ammonium, total phosphorus and available phosphate), microbial abundance, community-level physiological profiling, and soil enzymes (acid and alkaline phosphatases, dehydrogenase and urease) were investigated at two time intervals: zero-week (T0) and the eighth week (T8). Generally, microbial enumeration showed higher bacterial populations (496.63 × 10^4^ CFU g^−1^ dry soil) compared to actinomycetes (13.43 × 10^4^ CFU g^−1^ dry soil), and the fungal population was the lowest (67.68 × 10^2^ CFU g^−1^ dry soil) at T8. Functional diversity showed a strong, statistically significant positive effect in the G, N and S sites at T8. Acidobacteriota and Actinobacteriota declined in most places, while Firmicutes, Crenarchaeota and drought-tolerant bacteria such as Gemmatimonadota exhibited resistance. The fungal communities showed site-specific responses, with an increase in drought-tolerant Mortierellomycota and Chytridiomycota and a decrease in Ascomycota and Basidiomycota, suggesting possible adaptability. Overall, the microbial populations, enzyme activity, and functional diversity were positively correlated with soil moisture content across all four investigated sites. The significance of organic matter, soil structure, and moisture retention in determining microbial resilience to drought is underscored by these changes in microbial diversity and function, which in turn affect nutrient cycling and soil ecosystem stability. The findings of our study indicate that soil biological activities in agricultural regions can be modified by a mere two months of drought.

## 1. Introduction

Drought is one of the adversities that result in significant agricultural losses (defined as water scarcity). A future decline in rainfall is anticipated and may become worse due to a rise in temperature, leading to more frequent drought events [1]. Climatic predictions show more frequent drought events and may become more severe, especially during the summer season [2]. In recent years, Europe has experienced an increase in the frequency of prolonged dry conditions [3]. The agricultural ecosystem is so susceptible to drought stress that it may jeopardize global food security [4]. Generally, social and economic indices such as economic loss and crop production loss are employed to quantify drought-related losses. However, studies conducted on the effect of drought on the resilience of the soil ecosystem are still lacking. Therefore, it is crucial to use appropriate indicators to determine the effect of drought on agricultural soil and the soil ecosystem’s resilience [4].

Interactions between global change drivers and soil communities remain unclear [5,6]. A crucial environmental component in the metabolism of microorganisms is the natural variation in soil moisture due to seasonal variations and precipitation [7]. Drought conditions alter the variety and composition of the soil’s microbial community and the rate of mineralization [5]. Moisture in the soil is necessary for the movement of microbes, diffusion of substances between living cells, and hydrolysis processes [8]. The impacts of drought on soil quality are so severe that water availability is the primary limiting factor in soil biological activity [8]. There is mounting evidence that microbial activity is sensitive indicators as they directly reflect ecosystem stability and fertility to environmental stresses such as drought [9,10]. According to [11], reduced connections between soil’s functional microbial communities were the outcome of drought stress. According to the study conducted by [6], compared to fungal communities, bacterial diversity and composition showed a higher susceptibility to drought stress. In addition, according to [12], severe drought reduced gram-positive bacterial populations (−15%) and soil respiration (−35%). Soil microbial communities are susceptible to drought, which can limit their access to resources due to desiccation, reduced substrate supply and diffusion [9]. Hence, low soil moisture levels can reduce microbial activity, such as nutrient mineralization, enzymatic activity, and dormancy, allowing microbes to focus on their survival rather than multiplication [1,9]. As the most prevalent type of soil microorganism, bacteria are essential for overall soil health, and variations in soil moisture levels may directly impact their physiological state and may reduce their ability to break down resources (e.g., organic substrates). Starvation, along with induced osmotic stress and resource competition that can occur during periods of moisture limitation may impact on bacterial populations, exerting substantial selective pressure on their form and function [13].

Drying is uneven and can cause a confined drought that affects microorganisms, especially in bigger pores of soil aggregates (the primary habitat of microbial communities) [14]. A reduction in pore affinity may restrict bacterial movement and substrate diffusion, thereby altering the composition of the microbial communities [14,15]. On the other hand, fungi appear to survive in dry conditions more successfully than bacteria and generally remain unaffected by desiccation [16]. In addition, the phytopathogenic fungi could also thrive in such conditions [15]. As a result, the question arises whether variation in soil moisture content influences microbial communities and whether bacterial and fungal communities are sensitive or resistant.

Extracellular enzymes are produced and secreted by soil microorganisms and play a significant role in the soil matrix [15,17]. Enzymatic activity in the soil has been proposed as a potential sensitive biomarker of soil quality changes [15,18]. The enzymes that regulate nutrient availability and soil fertility can be affected by factors affecting soil microbial activity, as they play a significant part in soil nutrient cycling [15,17]. As a result, lower enzyme activity caused by drought may have a detrimental impact on enzyme structural integrity and nutrient availability, thereby jeopardizing the existing structural integrity of the enzyme [18]. Therefore, microbial biomass, community composition, metabolic activity, functional diversity, and numerous enzyme activities are frequently examined to understand the subtle changes in soils [19,20,21].

BIOLOG assays have been used as indicators of potential soil microbial communities in the utilization of a diverse range of carbon substrates. Evaluating the capacity of soil microbial communities to metabolize a variety of various organic carbon substrates with varying degrees of structural complexity is beneficial. This forms the basis of microbial community-level physiological profiles (CLPPs) (BIOLOG), which characterize the metabolic diversity of environmental samples [22]. This method can provide an extensive dataset that is suited for identifying site-specific variations in soil microbes and assessing the link between biodiversity and site conditions. The susceptibility of redox dye to temperature, its inefficiency on aquatic samples, and its effectiveness on heterotrophic bacteria are some of its drawbacks. Metabolic or physiological diversity, respiration activity and taxonomic diversity are very important factors, which help estimate the impact of drought on microorganisms [22,23].

In this study, we also performed non-redundant analysis as the dynamic interplay between microbially driven processes (such as carbon cycling and enzyme activity) and abiotic elements (such as pH and nutrients) are essential to soil ecosystems. For ecosystem health and sustainable land management, it is essential to comprehend the intricate interactions between biological activities and soil physicochemical characteristics. To overcome this difficulty, non-redundant analysis separates the overlapping effects of variables that frequently show multicollinearity in conventional evaluations, such as pH, organic carbon, nutrient availability, and microbial activity. Researchers can identify the distinct contributions of specific components, such as how pH controls the kinetics of enzymes (such as phosphatases) or how organic carbon propels microbial growth, by using approaches such as variance partitioning and redundancy analysis (RDA) [24]. The ability of RDA to identify non-intuitive drivers is demonstrated by studies on the coexistence of *Trichoderma* species, which showed that the available zinc content, even though it did not have direct inhibitory effects, was the strongest predictor of fungal distribution when analyzed alongside 26 soil parameters [25]. Similarly, soil moisture gradients and texture variations often overshadow biological responses unless analyzed through constrained ordination methods [26]. Non-redundant frameworks avoid misunderstandings linked to correlated variables by differentiating between direct and indirect effects, such as how urease activity is regulated by ammonium availability or how clay content affects nutrient retention. This method is especially crucial in agricultural systems, where precision targeting relevant components is necessary to balance carbon-to-nitrogen ratios for effective mineralization or optimize pH to minimize aluminum toxicity [27]. Recent developments in Monte Carlo validation and permutation testing significantly improve these models’ dependability and guarantee the reliable identification of important soil health indicators [28,29]. Theoretical ecology and real-world land stewardship are ultimately connected by non-redundant analysis, which offers useful insights for improving nutrient cycling, repairing damaged soils, and forecasting microbial reactions to environmental change [30,31]. Non-redundant analysis avoids the multicollinearity traps in predictive modelling by deciphering these intricate linkages to find distinct drivers of soil health. Non-redundant analysis separates the overlapping effects of physicochemical and biological elements to identify distinct drivers of soil ecosystem performance. By highlighting separate relationships and reducing multicollinearity, this method makes precise management techniques possible.

This work aims to assess the impact of an induced drought (2 months) on four different agricultural soil samples (based on soil bonitation classification (G (first class), L (third class), N (third class), S (fifth class)). We hypothesized that drought stress would cause changes in microbial diversity, enzymes, functional diversity, and physicochemical parameters. This research is the continuation and a part of the extensive body of research on drought that has already been published during other seasons (spring and autumn). In order to achieve these objectives, both physicochemical (pH, organic carbon (C), calcium carbonate (CaCO_3_), total nitrogen (N), nitrate (NO_3_^−^), ammonium (NH_4_^+^), total phosphorus (P), available phosphorus (P_2_O_5_)) and specific biological parameters (such as microbial diversity, CLPP, and soil enzymes (phosphatases (acid; ACP and alkaline; AKP), dehydrogenases (DH), ureases (UR)) were evaluated in stressed soils. We also conducted statistical analysis that compares the other most relevant variables between the biological, physicochemical and amplicon sequence datasets to see the joint effects, which is the novelty of this study. Moreover, it is still unknown whether the survival or existence of microbial community is totally dependent on a specific soil water content [1,32].

## 2. Materials and Methods

### 2.1. Soil Sampling and Chemical Analysis

**Figure 1:**
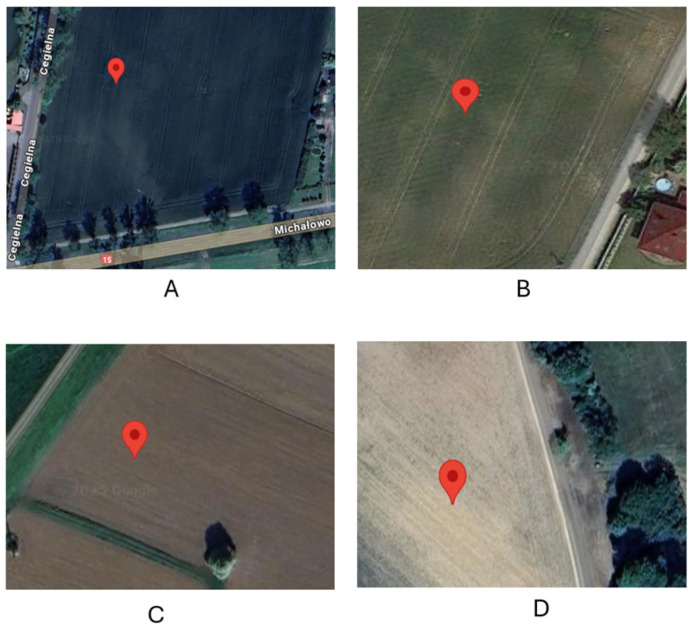
The map showing the investigated four locations at (**A**): Gniewkowo (G); (**B**): Lulkowo (L); (**C**): Wielka Nieszawka (N); and (**D**): Suchatówka (S).

In this study, we investigated four agricultural soil types. They ranged from gleyic luvisol (or luvic gleyic) Phaeozem in Gniewkowo (G; 52.901355° N, 18.432330° E), stagnic luvisol in Lulkowo (L; 53.090471° N, 18.581886° E), and fluvisol in Wielka Nieszawa (N; 53.006132° N, 18.466123° E), to haplic luvisol in Suchatówka (S; 52.907913° N, 18.468645° E), (Figure 1), located near Toruń, Poland. For each site, soil samples were collected in five plastic barrels (with the following dimensions: height = 23 cm, Ø = 28 cm, and V = 10 dm^3^) at a depth of 20 cm from the soil surface for the 0, 1st, 2nd, 4th, and 8th week treatments (henceforth referred as T0, T1, T2, T4, and T8, respectively), conducted during the summer season on 18 July 2022. A total of 10 soil samples with a volume of 1 dm^3^ from a given site were transferred to each barrel, which amounted to 10 dm^3^ in total. In total, 20 plastic barrels (5 per site) were subjected to induced drought conditions by placing them outside, under a shelter (protected from rainfall), for 2 months at an ambient temperature. A stainless-steel soil sampler probe (Ø 50 mm) was used to collect the samples at each time interval and directly subjected to further analysis in triplicate. The average soil moisture was determined using the gravimetric technique (samples dried at 100 °C for 4 days). The experiments were carried out in three replications, under laboratory conditions, previously passed through a 2 mm mesh sieve. Soil pH was measured in distilled deionized water, in soil—solution ratios of 1:2.5 using a pH meter CP-401 (ELMETRON, Zabrze, Poland). Total carbon (TC) and total nitrogen (TN) were determined using the organic elemental analyzer Vario Macro Cube (Elementar Analysensysteme GmbH, Langenselbold, Germany). Total phosphorus (P) was determined by the Bleck method, as modified by [33], and measured colorimetrically on a Rayleigh UV-1601 spectrophotometer. Available phosphorus was determined using the Olsen method [34] colorimetrically on a Rayleigh UV-1601 spectrophotometer and converted to P_2_O_5_ (available phosphorus). Calcium carbonate (CaCO_3_) was determined by the volumetric method using the Scheibler apparatus [35]. Total inorganic carbon (TIC) was calculated from the calcium carbonate content, and total organic carbon (TOC) was calculated from the difference between TC and TIC. Nitrate nitrogen [N-NO_3_] and ammonium nitrogen [N-NH_4_] were determined in the aqueous extract in a soil–water ratio of 1:2.5 [34] using the colorimetric method on the Merck Spectroquant Prove 100 spectrophotometer using Merck test kits. The texture and graining of the soil were determined according to the Bouyoucos areometric method modified by Casagrande and Prószyński [36] and the sieve method [35].

### 2.2. Enumeration of Microorganisms

A culture-dependent analysis was performed in triplicates at time (T) intervals 0, 1, 2, 4 and 8 weeks, where “0” is the sampling day. Bacteria, actinomycetes, and fungi were enumerated using a standard ten-fold dilution plate procedure for the four sites. About 10 g of fresh soil (in triplicates) were mixed into 90 mL of sterile physiological water for 10 min. An aliquot of decimal dilutions ranging from 10^−2^ to 10^−6^ was carried out. Bacterial abundance was determined by pouring 1 mL of 10^−4^, 10^−5^, and 10^−6^ decimal dilutions onto plate count agar (Table S1) (PCA agar, Biomaxima) in triplicates supplemented with cycloheximide (0.1 g L^−1^) to prevent fungal growth. Fungal abundance was performed by spreading 0.1 mL of the 10^−2^, 10^−3^, and 10^−4^ decimal dilutions in triplicate on the surface of Rose Bengal agar (Table S1) (Biomaxima), supplemented with chloramphenicol (0.1 g L^−1^) to prevent bacterial growth. The actinomycetes abundance was counted by surface spreading 0.1 mL of the 10^−3^, 10^−4^, and 10^−5^ decimal dilutions in triplicate on the actinomycete isolation agar (Table S1) (Becton Dickinson), supplemented with cycloheximide (0.1 g L^−1^) to prevent fungal growth. All Petri dishes were then incubated at 28 °C for 14 days. The number of culturable microorganisms is expressed as log10 of colony-forming unit (CFU) per gram of drought soil.

### 2.3. Characterization of Bacterial (16S) and Fungal (ITS) Diversity, and Bioinformatic Analysis

The total soil bacterial and fungal DNA extraction at T0 and T8 were extracted, and all PCR reactions were carried out at Novogenes (Cambridge, UK) using the CTAB method. The amplification of 16S rRNA/ITS1 genes of distinct regions (16S V3-V4/ITS1) was performed using a specific primer (515F-806R/ITS1). All PCR reactions were conducted using 10 ng of template DNA, 2 μM of forward and reverse primers, and 15 μL of Phusion^®^ High-Fidelity PCR Master Mix (New England Biolabs, Hitchin, UK). The thermal cycling process included a one-minute initial denaturation at 98 °C, 30 cycles of denaturation at 98 °C for 10 s, annealing at 50 °C for 30 s, and elongation at 72 °C for 30 s and 72 °C for 5 min. The combined PCR products with the same volume of 1X loading buffer (which includes SYB green) were then subjected to electrophoresis on a 2% agarose gel for detection. Equidensity ratios were used to combine PCR products. Following that, th emixed PCR results were purified using Qiagen Gel Extraction Kit (Qiagen, Hilden, Germany). The library quality was assessed on the Qubit@ 2.0 Fluorometer (Thermo Scientific, Cambridge, UK), an Agilent Bioanalyzer 2100 system and sequenced on an Illumina NovaSeq platform. The generated paired-end sequences, in FASTQ format and all the downstream analyses at T0 and T8 for 16S and ITS sequence were performed using DADA2 pipeline in R (v4.3.2) within R studio. We initiated data sequencing quality control for 16S and ITS reads using DADA2 pipeline in R (v4.3.2). This involved removing chimeric sequences and identifying Amplicon Sequence Variants (ASVs). Subsequently, the SILVA v138.1 and UNITE databases were utilized for taxonomic assignment for 16S and ITS reads, respectively.

### 2.4. Soil Enzymatic Activities

Enzymatic activities were determined spectrophotometrically in triplicate for all four investigated soil samples. Dehydrogenase activity (DH) was measured according to [37] by quantifying the triphenylformazan (TPF) obtained after the incubation of 1 g of fresh soil (0.8% in 0.1 M Tris-HCl buffer pH 7) at 37 °C for 24 h. The obtained TPF was extracted with acetone (100%) and absorbance was measured at 490 nm against the blank (prepared as above without TTC) using a Marcel Pro Eko spectrophotometer (Poland). The alkaline (AKP) and acid (ACP) phosphatase activities were determined according to [37], using ρ-nitrophenyl phosphate (ρ-NPP) as a substrate after 4 h of incubation at 30 °C. The amount of sodium p-nitrophenylphosphate (PNP) was determined by measuring the absorbance at 410 nm using a Marcel Pro Eko spectrophotometer (Poland). Urease activity (UR) was measured according to the methodology presented by [38] by spectrophotometric determination of the amount of ammonium produced. Optical density was measured against the blank at 420 nm using a Marcel Pro Eko spectrophotometer (Warsaw, Poland).

### 2.5. Metabolic Diversity

The impact of induced drought was evaluated on microbial diversity in the investigated soil samples, collected at T0, T1, T2, T4, and T8, using metabolic profile assessment, respectively. For this, Biolog EcoPlates (Biolog Inc., Hayward, CA, USA), containing 31 different carbon sources (including a blank well) in triplicate, were used. A total of 100 µL of 10^−2^ decimal dilutions of soil microbial suspension was directly inoculated in the plates. All plates were incubated at 28 °C for 96 h, and absorbance was measured using a Multiskan FC photometer microplate reader (Thermo Fisher Scientific, Waltham-MA, USA) at 590 nm. The average well color development (AWCD) was evaluated using the method described by [23].

### 2.6. Statistics

The microbial abundance data were log-transformed as the relative abundance of each CFU/mL within a sample site. All soil biological and physicochemical parameters were measured (three repetitions) and statistically analyzed using a two-way analysis of variance test (ANOVA) at the 0.05 confidence level and a Tukey test. The declared level of significance is *p* < 0.05. The calculation of the correlation matrix between all chemical and biological criteria was determined using Pearson coefficients. A two-way ANOVA was applied to the principal component analysis (PCA) factors to determine the significant differences between biological and physicochemical parameters with respect to soil moisture content. It was also used to analyze the CLPP data. The bar plots, box plots, heatmaps, correlation analysis, and PCA (in triplicate) results were visualized using R software (V.4.3.2) with the “ggplot2” package [39]. The amplicon sequences obtained were rarified, and the statistical analyses were also conducted in R Studio using the phyloseq packages and ggplot2 [40]. Bacterial and fungal alpha diversity indices and their plots (Shannon and Simpson) were also calculated in R [41].

## 3. Results

### 3.1. Chemical Properties of Soil Samples

**Figure 2:**
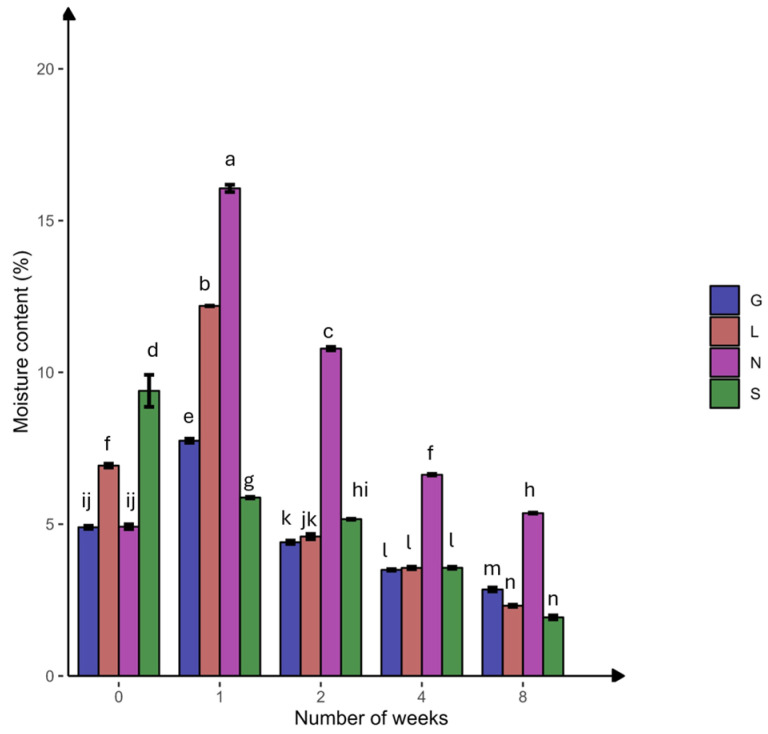
Percentage of soil moisture content at four agricultural soil samples collected from Gniewkowo (G), Lulkowo (L), Wielka Nieszawka (N), and Suchatówka (S) (*p* value < 0.05). All statistical analyses were carried out using two-way ANOVA and Tukey’s test at *p* < 0.05. Different lowercase letters indicate significant differences.

**Figure 3:**
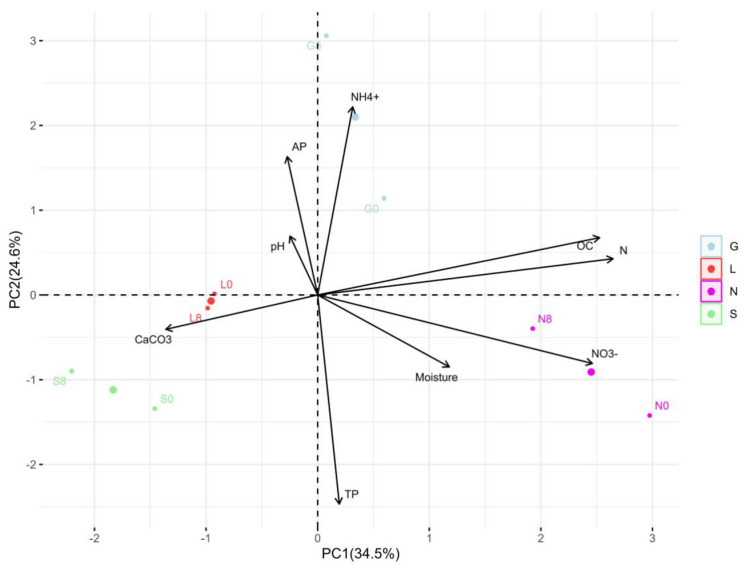
Principal component analysis (PCA) for the distribution of each site, as scattered among the first two components of the PCA (95% confidence ellipses), and respective grouping in terms of their correlations between the soil moisture content and physicochemical parameters at T0 and T8 at investigated sites. G: Gniewkowo, L: Lulkowo, N: Wielka Nieszawa, S: Suchatówka, 0: week 0, 8: week 8.

In this study, the N site had the highest clay content, but the S site had the lowest clay content compared to other sites (Table 1). The silt fraction decreased in the S site but increased in other sites at T8. The highest moisture content was at T1, followed by a significant decline toward the end of the experiment, with the highest reduction in the sandy S site (Figure 2). The carbon and nitrogen contents significantly decreased (*p* < 0.05) at the N site (Table 2). At T8, a significant difference (*p* < 0.05) was observed for P, P_2_O_5_, and NO_3_^−^ at all sites. The S site was more calcareous compared to the other sites and decreased significantly (*p* < 0.05) at T8, while remaining constant at the other sites. The NH_4_^+^ content decreased and increased significantly (*p* < 0.05) at the G and S sites, respectively. The average total phosphorous content was higher compared to the available phosphorus (P_2_O_5_). The pH ranged from slightly acidic to alkaline conditions. Overall, Table S2 confirms the significant positive correlation (r = 0.179–0.994 range; except for CaCO_3_, at the L site, which was not applicable due to zero CaCO_3_ content) between the soil moisture content and physicochemical properties at T0 and T8. The pH showed a strong positive correlation with calcium carbonate (G0: 0.99, G8: 0.89) (Table S7). Phosphorus showed a strong positive correlation with available phosphorus (G8: 1.00). Nitrogen showed a strong positive correlation with nitrate (G8: 0.98, L8: 1.00, N8: 0.99) (Table S7 and Figure 3). The PCA analysis (Figure S1A) revealed that a major portion of the total variance (97.5%) of the studied variables (soil physicochemical parameters) was grouped between five components, and two of them explained 59.1% (Figure S1B). The variables that contributed most to Dim1 of the PCA axes were OC (0.93), N (0.97), TP (0.07), and NO_3_^−^ (0.91), whereas NH_4_^+^ (0.82) and AP (0.60) contributed most to Dim2 (Figure S1A). The results presented in Figure 3 reveal a clear and statistically significant positive significance between sites (G, L, N, S at T0 and T8) and the soil physicochemical parameters with respect to the soil moisture content. More specifically, the G soil site was scattered in the upper right–centered quadrant of the PCA plot, indicating a strong association with OC, N, AP, pH, and NH_4_^+^ (Figure 3). On the contrary, the L and S sites were scattered in the lower left–centered quadrant, showing an association with AP, pH, and CaCO_3_ (Figure 3). A corresponding association was observed for the N site, which was scattered in the lower right quadrant with NO_3_^−^, TP, OC and N variables, respectively (Figure 3).

### 3.2. Biological Parameters

#### 3.2.1. Microbial Concentration

**Figure 4:**
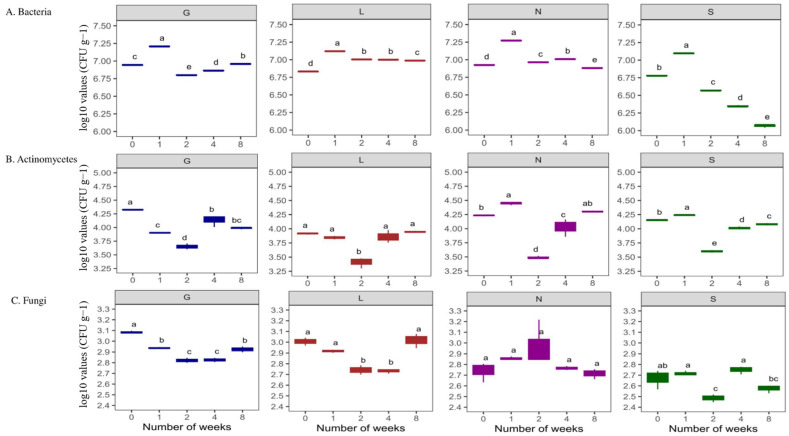
Changes in number of microbial populations under varying soil moisture conditions across four agricultural soil samples. All analyses were performed in triplicate, and the data are presented as mean ± SD. All statistical analyses were carried out using a one-way ANOVA and Tukey’s test at *p* < 0.05. G: Gniewkowo, L: Lulkowo, N: Wielka Nieszawa, S: Suchatówka, 0: week 0, 8: week 8. Different lowercase letters indicate significant differences.

**Figure 5:**
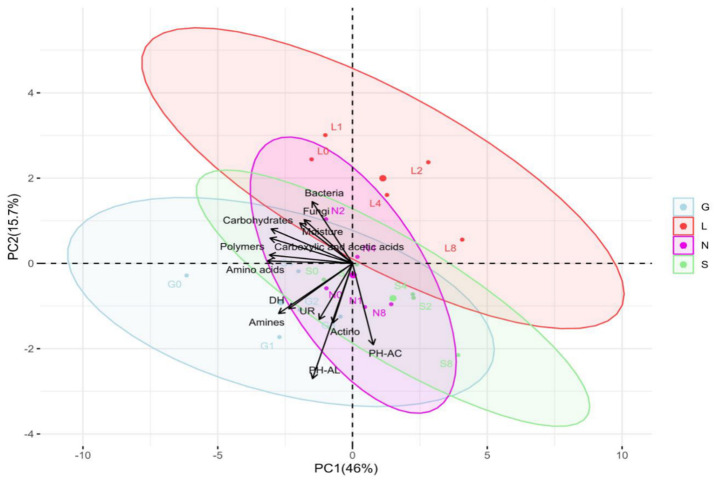
Principal component analysis (PCA) indicating correlations between soil moisture content and biological parameters across four soil samples at T0 and T8 time intervals. The distribution of each site is scattered among the first two components of the PCA (95% confidence ellipses) and respective grouping. G: Gniewkowo, L: Lulkowo, N: Wielka Nieszawa, S: Suchatówka, 0: week 0, 8: week 8, Actino: Actinomycetes, PH-AC: Acid phosphatase, PH-AL: alkaline phosphatase, DH: dehydrogenase, UR: urease, 0: week 0, 1: week 1, 2: week 2, 4: week 4, 8: week 8.

This study showed higher bacterial abundance (496.63 × 10^4^ CFU g^−1^ dry soil) compared to actinomycetes (13.43 × 10^4^ CFU g^−1^ dry soil), and fungal abundance was the lowest (67.68 × 10^2^ CFU g^−1^ dry soil) abundance (Table S3). Site G had the highest bacterial counts (648.67 × 10^4^ CFU g^−1^ dry soil), and lowest was at S and L sites (approximately 356 × 10^4^ CFU g^−1^ dry soil) respectively (Table S3). The N site had the highest actinomycetes counts (17.27 × 10^4^ CFU g^−1^ dry soil) and the L site had the lowest (8.14 × 10^4^ CFU g^−1^ dry soil) (Table S3). In the case of fungal counts, the G site had the highest counts (84.93 × 10^2^ CFU g^−1^ dry soil) and the S site the lowest (44.80 × 10^2^ CFU g^−1^ dry soil) (Table S3). Although bacterial populations were higher at T1, when soil moisture content was at its highest, their abundance decreased significantly (*p* < 0.05) at T8 at all sites (drastically at the S site), along with a decrease in soil moisture content (Figure 4A). In the case of Actinomycetes, in general they were stable (Figure 4B) at T8 compared to T0 (*p* < 0.05; G and S sites). Fungal abundance was lowest compared to bacteria and actinomycetes (Figure 4C). A decreasing trend in fungal population was observed at all sites at T8 compared to T0, significantly for the G site (*p* < 0.05) (Figure 4C). In general, this study shows the decrease in bacterial abundance was faster and stronger (*p* < 0.05) compared to fungal abundance (Table S3 and Figure 4). Overall, Table S4 confirms a significant positive correlation (r = 0.15–0.99 range) between the soil moisture content and microbial abundance at T0 and T8. In addition, Table S5 reveals an overall positive correlation between microbial abundance and enzyme activities at T0 and T8. The PCA analysis (Figure 2 and Figure S2A) revealed that the major portion of the total variance (87.7%) of the studied variables was grouped between five components, and the two of them explained 61.7% (Figure S2B). Bacteria (0.44) and fungi (0.32) contributed to the Dim1 of PCA axes, whereas actinomycetes (−0.22) contributed mostly to Dim2 (Figure 2 and Figure S2A). The results presented in Figure 5 reveal a clear and statistically significant positive difference between the sites (G, L, N, S at T0 and T8) and microbial abundance with respect to the soil moisture content. More specifically, the G site showed a strong association with the abundance of actinomycetes. On the contrary, the L site showed strong significance with bacteria and fungi (Figure 5). A corresponding strong association was observed for N and S sites indicated a strong significance with bacteria, actinomycetes and fungi (Figure 5).

In non-independent correlation analysis (Table S7), bacterial abundance showed strong correlations with nitrate at G0, L8, N8, and S8 (1.00) and soil moisture at G8: 0.77. Actinomycetes, showed a strong positive correlation with acid phosphatase at G8: 0.99; L8: 0.91; N8: 0.95; and S0: 0.94. Soil moisture showed a strong positive correlation at G8: 0.76; L8: 0.83; and N8:0.98.

#### 3.2.2. Enzyme Activities

**Figure 6:**
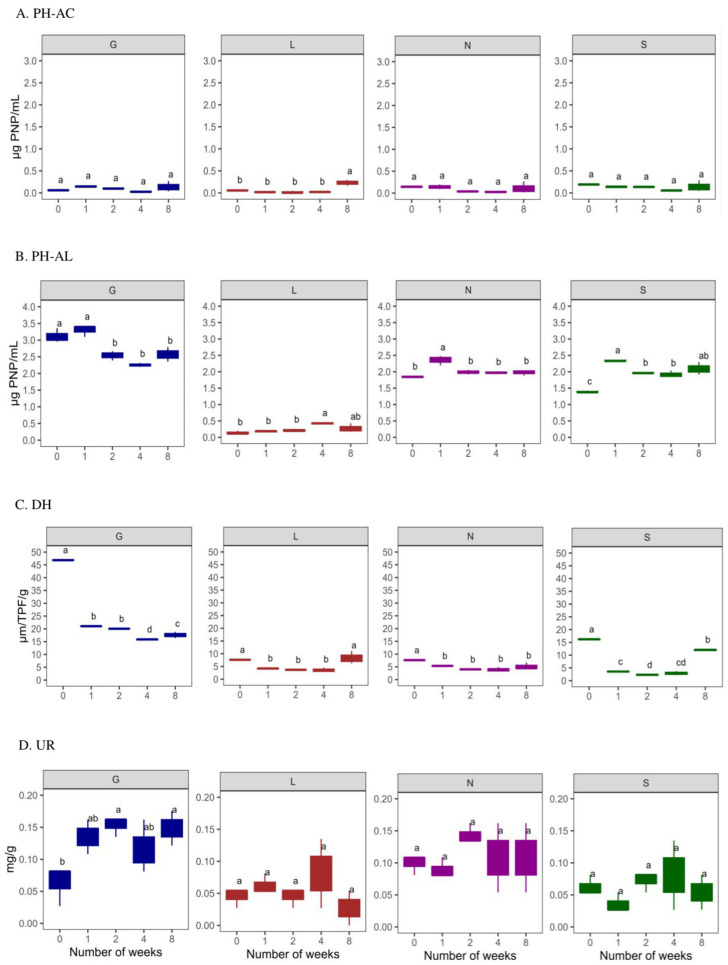
Enzyme activities at four types of agricultural soil samples. (**A**) Acid phosphatase (ACP); (**B**) alkaline phosphatase (ALP); (**C**) dehydrogenase (DH) (**D**) urease (UR) enzyme activities. All analyses were performed in triplicate, and the data are presented as mean ± SD. All statistical analyses were carried out using one-way ANOVA and Tukey’s test *p* < 0.05. G: Gniewkowo, L: Lulkowo, N: Wielka Nieszawa, S: Suchatówka. Different lowercase letters indicate significant differences.

The induced drought conditions negatively affected AC (6.25% lower)to a greater extent than AL (93.49%) activity (Figure 6A,B). The sandy S site had the highest AC, whereas AL activity was highest at the G site. The AC activity was lowest at T0 compared to T8 under drought conditions. The L site was observed to have the lowest AC (*p* < 0.05) and AL activities. In the case of AL, its activity increased at T8 of our experiment. The DH activity was highest at T0 and decreased significantly at T8 (*p* < 0.05; except for the L site), with a decrease in the soil moisture content at all sites (Figure 6C). In addition, the UR activity was lower at T0 compared to T8 (*p* < 0.05 for G site) (Figure 6D). The G site recorded higher enzymatic activities, followed by the N, L and S sites. In addition, PCA analysis confirms the positive significance of soil moisture content with ACP and DH activities (Figure 5). Overall, Table S4 confirms a significant positive correlation (r = 0.02–0.99 range; except for UR at the N8 site) between the soil moisture content and enzymatic activities at T0 and T8. The PCA analysis (Figure S2A) revealed that a major portion of the total variance (87.7%) of the studied variables (PH-AC, PH-AL, DH, and UR) was grouped between five components, and the two of them explained 61.7% (Figure S2B). The PH-AC (0.23), PH-AL (−0.45) and UR (−0.37) contributed to Dim1 of the PCA axes, whereas DH (−0.32) contributed mostly to the Dim2 (Figure S2A). The results presented in Figure 5 show a statistically significant positive difference between the sites (G, L, N, S at T0 and T8) and the enzymatic activities with respect to the soil moisture content. More specifically, the G, N, and S sites showed a strong association with the investigated enzymatic activities. The correlation analysis between the non-independent samples (Table S7) showed a strong positive correlation between pH and alkaline phosphatase at L8: 0.98, N8: 0.99, and S8: 0.9. The available phosphorus showed a strong positive correlation with alkaline phosphatase at N8:0.98. Phosphorus showed a strong positive correlation with alkaline phosphatase at G8: 0.99, L8: 1.00, S8: 0.99 and a moderate correlation with N8: 0.93. Soil moisture showed a strong positive correlation with alkaline phosphatase at G0: 0.96; L0: 0.99, and a weak correlation with urease at G8:0.02.

#### 3.2.3. Analysis of Soil Microbial Communities Using Community-Level Physiological Profiles (CLPPs)

**Figure 7:**
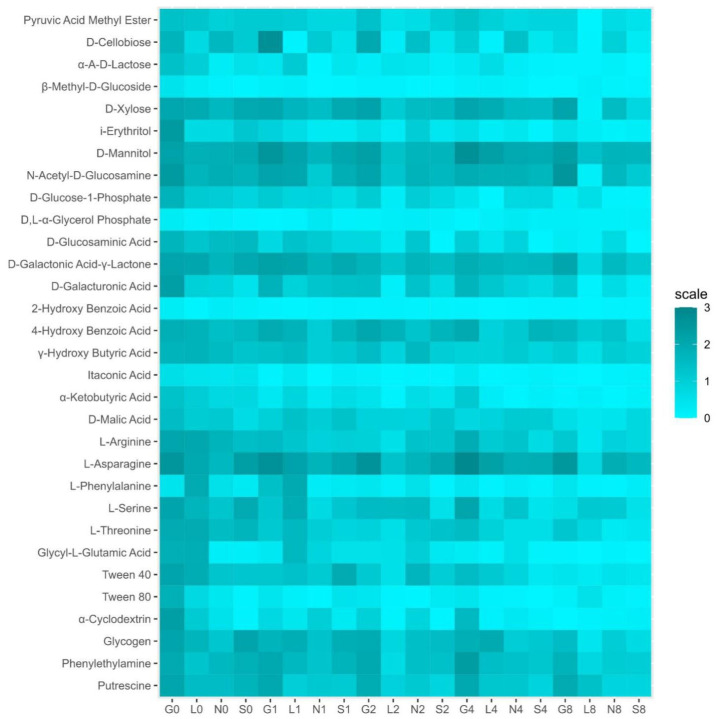
Heat map for community level physiological profiles (CLPPs) in four types of agricultural soil samples. G, Gniewkowo; L, Lulkowo; N, Wielka Nieszawa; S, Suchatówka.

The Biolog EcoPlate includes five major groups of carbon sources, mainly carbohydrates (CH), carboxylic, and acetic acids (CAs), amino acids (AAs), amines (AM), and polymers (PL). The average well color development (AWCD) declined from T1 to T8 (Figure S3A). The G site had the highest metabolic rate compared to other sites (Figure S3B–F and Figure 7). On the contrary, the L site showed low utilization of CA, AA, and amines, whereas the S site showed the lowest utilization of CH and PL (Figure S3B–F and Figure 7). Overall, the rate of carbon source utilization decreased at all sites T8. Table S6 reveals a positive correlation (r = 0.11–0.98 range) between soil moisture and substrate decomposition activity of five major groups of carbon sources in all sites at T0 and T8.

The PCA analysis (Figure S3A) revealed that the major portion of the total variance (87.7%) of the studied variables (CH, CA, AA, AM, and PL) was grouped between five components, two of which explained 61.7% (Figure S3). All groups contributed to the Dim2 of PCA axes, i.e., CH (0.25), CA (0.06), AA (0.02), PL (0.18), and AM (−0.36) (Figure S3). The results presented in Figure 5 show a statistically significant positive difference between the sites (G, L, N, and S at T0 and T8) and the CLPP analysis with respect to the soil moisture content. More specifically, the G, N, and S sites showed a strong association with all five groups of carbon sources. On the contrary, the L site showed a statistically significant negative correlation only with amines (Figure 5). Generally, the breakdown of CLPP activities was observed at T8, whereas no changes or higher activity were observed at T0 (Figure S3).

According to the Shannon–Wiener index (H) (Table 3), total diversity decreased under stress (T8), especially at the L and G sites. Across all sites, T0 had the greatest diversity. Significant taxonomic loss under stress was noted in the case of richness. At T8, the number of taxa (richness) drastically declines, especially in L and G sites. In contrast to the L site, the S site maintains more richness. The slight decrease in T8 evenness across all locations indicates that, despite the loss of some species, the relative balance of the remaining species is preserved. The largest reduction in evenness is seen on the G site (Table 3).

While the N and S sites showed mixed patterns, the G and L sites exhibited a higher soil moisture correlation (Table S7). Carbon and nitrogen showed a strong positive correlation with microbial groups, whereas phosphorous correlated with microbial activity at the N and G sites. At T8, NO_3_^−^ showed mixed patterns at the N and S sites, but a strong positive correlation in the G and L soils (Table S7). The NH_4_^+^ correlation varied greatly, and under stress (such as at the N site), some showed a significantly positive effect. The wide range of pH correlations affected microbial diversity and enzymatic activities. Contrary to stress circumstances at T8, bacteria exhibited a greater association with NO_3_^−^, OC, and TP at T0 (Table S7). Overall, the fungal and actinomycetes response vary greatly based on the type of soil, and the N and G sites promote more stable microbial interactions. The CLPP analysis was observed to be associated with phosphate, OC, and soil moisture (Table S7).

The non-independent correlation analysis (Table S7) indicated strong a correlation between carbohydrates and carbon at G0 (1.00), L0 (0.87), N0 (0.83), and S0 (1.00). Polymers showed a strong positive correlation with calcium carbonate at the G8 and S (1.00) sites, and amino acids with ammonium at all sites. The available phosphorus showed strong positive correlation with polymers (S8: 1.00). Carbon showed strong correlation with amino acids (L8: 1.00, N8: 0.96, S0: 1.00), and soil moisture showed a strong positive correlation with polymers (S8: 1.00). A weak correlation was observed at the L and N sites. Overall, the most reliable positive associations were observed at the G site, indicating that it maintains microbial and enzymatic activity even under stressful conditions.

### 3.3. Microbial Composition and Diversity

**Figure 8:**
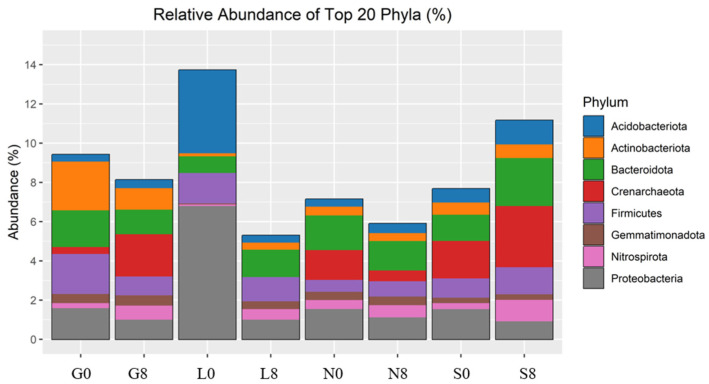
Relative abundance of bacterial phyla identified by 16S rRNA amplicon sequencing. G: Gniewkowo, L: Lulkowo, N: Wielka Nieszawka, S: Suchatówka, 0: collection date, 8: 8 weeks of drought.

**Figure 9:**
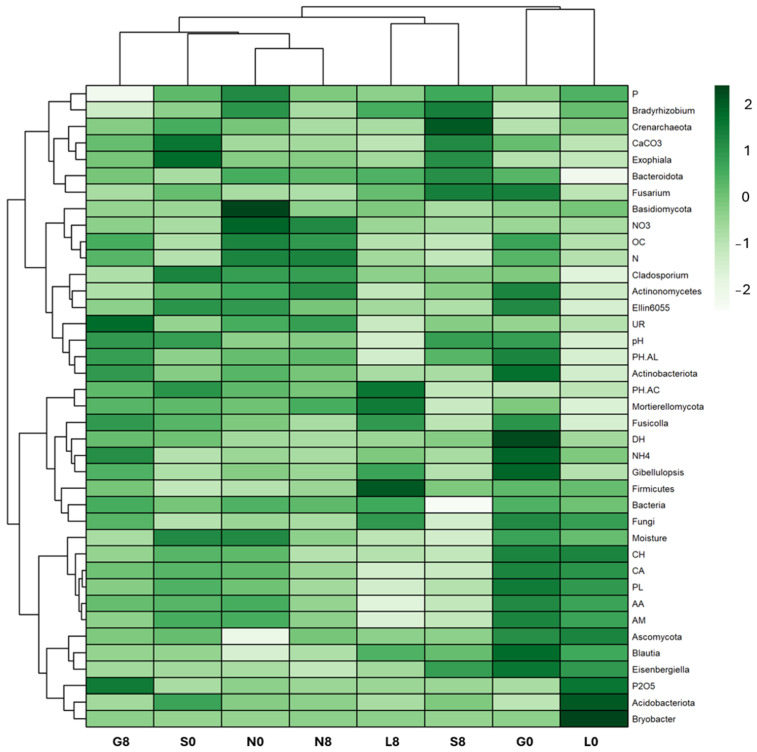
Soil cluster heatmap with most relevant variables between the biological, physicochemical, and amplicon sequence datasets to examine the joint effects. G: Gniewkowo, L: Lulkowo, N: Wielka Nieszawka, S: Suchatówka, 0: sampling day, 8: 8 weeks of drought; PH-AC: acid phosphatase, PH-AL: alkaline phosphatase, DH: dehydrogenase, UR: urease, OC: organic carbon, CaCO_3_: calcium carbonate, N: total nitrogen, NO_3_^−^: nitrate, NH_4_^+^: ammonium, P: total phosphorus, P_2_O_5_: available phosphorus, CA: carboxylic and acetic acids, CH: carbohydrates, PL: polymers, AA: amino acids, AM: amines, and PL: polymers.

**Figure 10:**
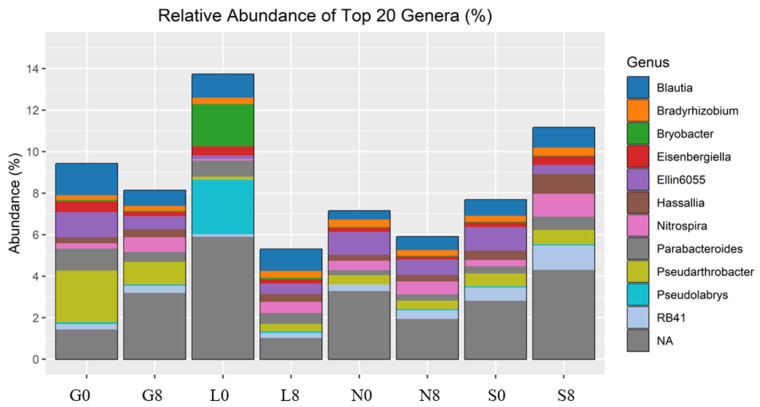
Relative abundance of bacterial genera identified by 16S rRNA amplicon sequencing. G: Gniewkowo, L: Lulkowo, N: Wielka Nieszawka, S: Suchatówka, 0: sampling day, 8: 8 weeks of drought.

The investigated sites (G, L, N, S) reveal notable changes in key phyla consisting of Actinobacteriota, Bacteroidota, Proteobacteria, Firmicutes, Acidobacteriota, Crenarchaeota, Gemmatimonadota, and Nitrospirota (Figure 8; Table 4) and genera including *Blautia*, *Bradyrhizobium*, *Bryobacter*, *Eisenbergiella*, *Ellin6055*, *Hassallia*, *Nitrospira*, *Parabacteroides*, *Pseudarthrobacter*, *Pseudolabrys*, and *RB41* (Figure 9 and Figure 10; Table 5). Actinobacteriota decreased from 27.70% (G0) to 22.77% (G8) (Table 4) and from 19.49% (N0) to 16.42% (N8) (Figure 8). The relative abundance of Bacteroidota increased from 13.15% (L0) to 19.58% (L8), from 16.81% (S0) to 21.36% (S8), and from 19.40% (G0) to 18.31% (G8). A consistent decrease in Proteobacteria was observed from T0 to T8. For example, at the L site, their abundance decreased from 27.18% (L0) to 17.58% (L8) and from 19.50% (N0) to 16.78% (N8). Firmicutes showed an increase in relative abundance from 13.00% (L0) to 21.01% (L8) (Figure 9) and from 7.80% (S0) to 11.95% (S8) (Figure 8; Table 5). At the G site, Acidobacteriota abundance increased from 6.35% (G0) to 8.77% (G8), while significantly decreasing from 26.23% (L0) to 7.93% (L8). Moderate and/or less significant results are indicated in the Supplementary File (see Results Section S3.2).

The relative abundance of *Blautia* decreased at the G (1.52% → 0.75%) and N (0.41% → 0.64%) sites (Figure 10; Table 4). *Eisenbergiella* declined across all sites, except for S soil (0.21% → 0.41%). *Ellin6055* decreased at the G (1.20% → 0.63%), N (1.11% → 0.74%), and S sites (1.14% → 0.44%). The relative abundance of *Bryobacter* decreased drastically at the L site (2.04% → 0.08%) (Figure 9). However, *Bradyrhizobium* was relatively stable across all conditions (Figure 9), indicating that this genus may be more resilient to drought stress (Figure 10; Table 4).

**Figure 11:**
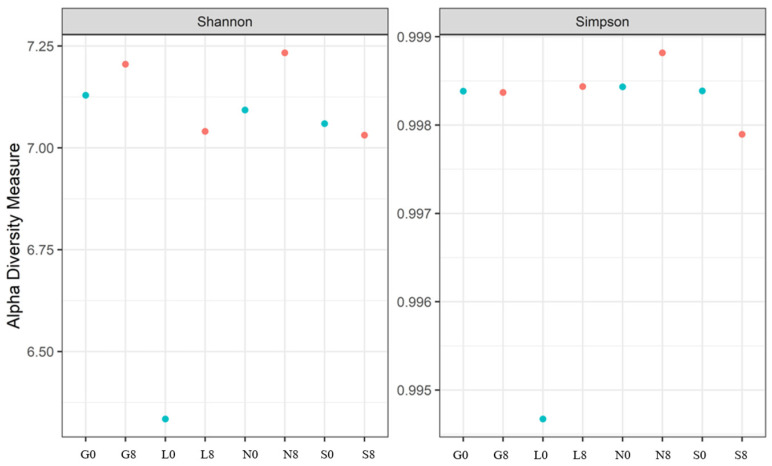
The Shannon and Simpson indices for the bacterial communities at four investigated sites. G: Gniewkowo, L: Lulkowo, N: Wielka Nieszawka, S: Suchatówka, 0: sampling day, 8: 8 weeks of drought.

Table 5 shows the bacterial alpha diversity indices, i.e., Shannon and Simpson, which suggest the evenness (distribution of species) and richness (number of species) of bacterial communities investigated at the T0 and T8 time intervals. Higher values of the Shannon index indicate greater bacterial variety, considering both species richness and evenness. The sites G, L, and N exhibit an increase in the Shannon index (Figure 11). A well-balanced bacterial population devoid of dominance by a single species is indicated by values near 1.00 on the Simpson diversity index, which gauges evenness. The fact that all values are approximately 1.00 indicates that the distribution of bacterial species was uniform (both before and after the drought) (Figure 11). The highest observed species richness and discrepancy between observed species and Chao1 estimations was observed at the G site. The species richness at N0, N8, S0, and S8 is underestimated, and the discrepancies between the estimated and observed values are negligible (Table 5). Soil moisture seems to have less impact on the Chao1 estimations (except for G0, where the difference is highly significant).

**Figure 12:**
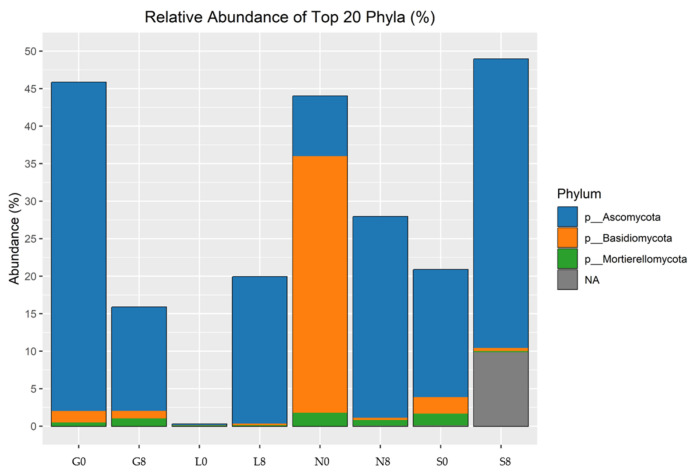
Relative abundance of fungal phyla identified by ITS amplicon sequencing. G: Gniewkowo, L: Lulkowo, N: Wielka Nieszawka, S: Suchatówka, 0: sampling day, 8: 8 weeks of drought.

Ascomycota is the most abundant fungal phylum across all sites, with values ranging from 48.98% to 81.67% (Figure 12; Table 6). Its abundance decreases over time at most sites, particularly at the G (13%), L (16.25%), and S (4.76%) sites, but increases at the N site (48.98% → 68.60%). Basidiomycota is the second most abundant phylum but shows major variation across the sites. Its abundance showed strong enrichment at N0 (Figure 9) but declined drastically at the N site (41.19% → 9.44%) and slightly declined at the G (1.12%), N (31.75%), and S (2.43%) sites.

**Figure 13:**
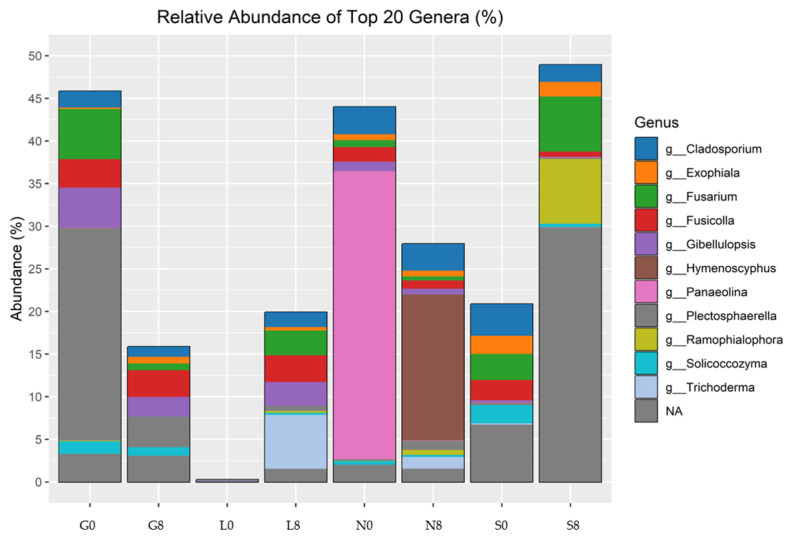
Relative abundance of fungal genera identified by ITS amplicon sequencing. G: Gniewkowo, L: Lulkowo, N: Wielka Nieszawka, S: Suchatówka, 0: sampling day, 8: 8 weeks of drought.

In terms of the relative abundance of fungal genra, *Cladosporium*, *Exophiala*, *Fusicolla*, *Gibellulopsis*, *Hymenoscyphus*, *Panaeolina*, *Plectosphaerella*, *Ramophialophora*, *Solicoccozyma*, and *Trichoderma* were observed (Figure 13; Table 6). *Gibellulopsis* showed a decrease at the G (2.37%), N (0.44%), and S (0.19%) sites but increased at the L (2.7%) site, whereas Fusarium abundance decreased at the G site (5.15%), but increased at the L (2.91%) and S (3.06%) sites. Although *Panaeolina* abundance decreased at all sites, it was prominent at the N site, with an abundance of 33.748%. *Cladosporium* and *Fusicolla* exhibited relatively stable abundance across drought conditions, but *Fusicolla* abundance increased at the L site by 3.12%. *Hymenoscyphus* showed a significant increase at the N site (17.1%).

**Figure 14:**
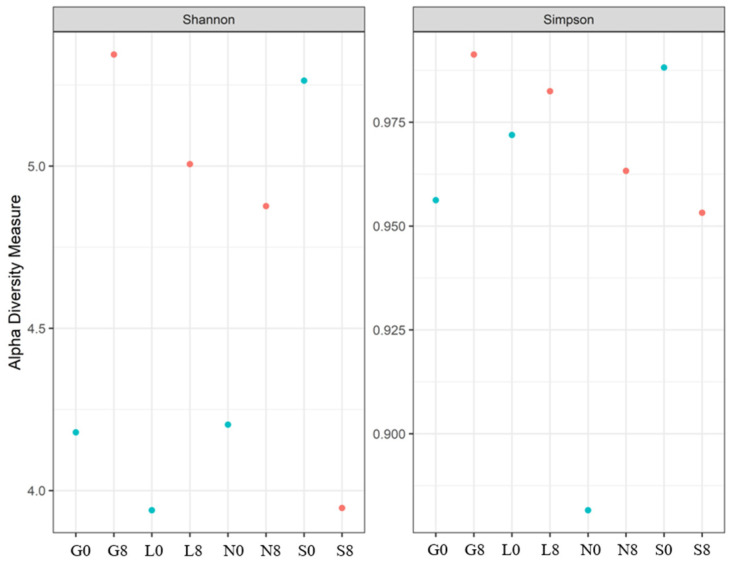
The Shannon and Simpson indices for the fungal communities at the four investigated sites. G: Gniewkowo, L: Lulkowo, N: Wielka Nieszawka, S: Suchatówka, 0: sampling day, 8: 8 weeks of drought.

A rise in fungal variety over the 8-week drought period is indicated by the Shannon index at the G, L, and L sites (Table 7; Figure 14), suggesting a balanced fungal community. The decline in the Shannon and Simpson indices at the S site increased the dominance of a few drought-resistant fungal communities but decreased the overall evenness. In the case of the Chao1 index, the highest diversity was observed at S0 (Table 7). A significant increase at L8 was observed, whereas S8 showed a sharp decline (47% loss).

**Figure 15:**
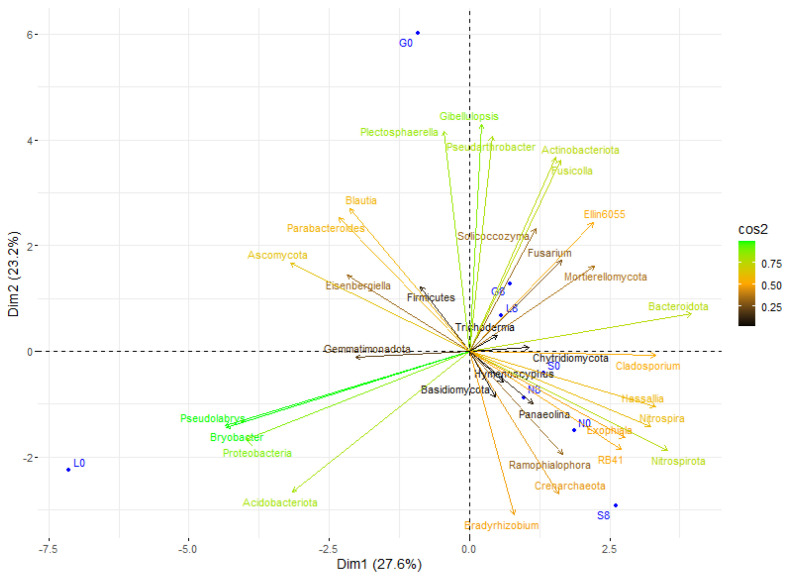
Principal component analysis (PCA) of the studied correlations in soil microbial community structure at T0 and T8 showed that each site was scattered among the first two components of the PCA (95% confidence ellipses). G: Gniewkowo, L: Lulkowo, N: Wielka Nieszawka, S: Suchatówka, 0: sampling day, 8: 8 weeks of drought.

Actinobacteriota and Pseudonocardia exhibited a positive correlation with drier conditions, particularly at L8 and G8 (Figure 15). In addition, Proteobacteria and Acidobacteriota showed a positive correlation at L0, indicating that they favor moist conditions. In addition, Firmicutes, *Fusicolla*, and Actinobacteriota may play a part in stress adaptation since they showed a positive correlation at G8 and L8 (Figure 9 and Figure 15). *Nitrospira* and Nitrospirota were positively correlated at N8 and S8, indicating elevated nitrification during stressful conditions. The fact that *Trichoderma*, *Plectosphaerella*, and *Solicoccozyma* are linked to stressed conditions (G8, S8) suggests that they play a part in decomposition and drought adaptation. Overall, the T8 samples were more centrally concentrated, which may indicate a decrease in microbial diversity and an increase in taxa that can withstand stress.

**Figure 16:**
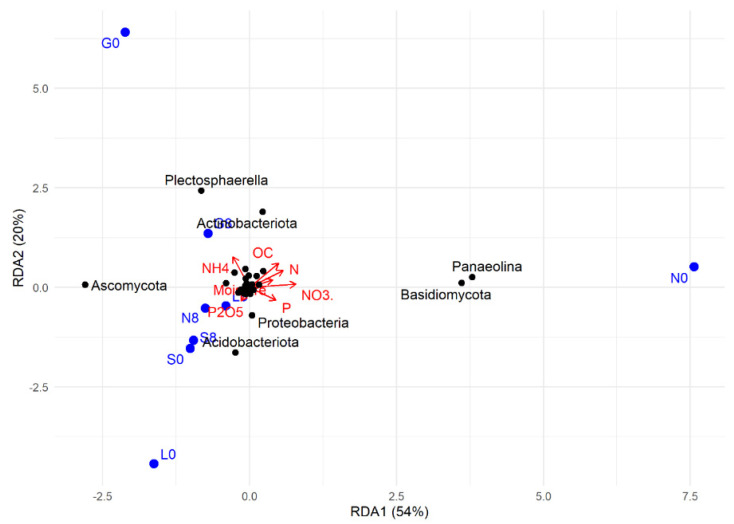
RDA (redundancy analysis) of the studied correlations between soil physicochemical factors and microbial community structure at T0 and T8, for each site. G: Gniewkowo, L: Lulkowo, N: Wielka Nieszawka, S: Suchatówka, 0: sampling day, 8: 8 weeks of drought; C: organic carbon, CaCO_3_: calcium carbonate, N: total nitrogen, NO_3_^−^: nitrate, NH_4_^+^: ammonium, P: total phosphorus, P_2_O_5_: available phosphorus.

The RDA plot shows how soil physicochemical variables influence microbial community composition (Figure 16). Among the sites investigated, most at T8 showed a strong positive correlation with OC, NH_4_^+^, N, *Plectosphaerella*, Actinobacteriota (G), and with P, P_2_O_5_, NO_3_^−^, Ascomycota, Proteobacteria, and Acidobacteriota at the L, N, and S sites (Figure 9 and Figure 16). At T0, the G, L, and N sites were mainly weakly associated with microbial taxa *Plectosphaerella*, *Panaelina*, and Acidobacteriota, respectively, and with soil physicochemical parameters.

## 4. Discussion

### 4.1. Soil Parameters

Clay surfaces may absorb more organic C molecules and lead to the formation of organo–mineral complexes. These complexes protect the soil organic carbon (SOC) from microbial decay, increasing SOC storage and water retention [42]. This explains the highest clay content, organic C, and soil moisture at the N site (15.99% at T1), followed by the G site, compared to the sandy S and L sites, which have lower water-holding capacity and organic C [42,43] (Table 1 and Table 2; Figure 2).

Soil drying affects nitrogen cycling differently than carbon cycling [9]. Drought limits diffusion of substrates such as NH_4_^+^, inhibiting nitrification, as seen at the N site, which exhibits low nitrogen at T8 (Table 2). Rewetting releases nitrogen from the microbial necromass, bacterial osmolytes, and nitrogen-rich clay-protected small molecules, causing temporary nitrogen pulses [9,32,44]. The Polish weather forecast aligns with sampling dates, explaining nitrogen fluctuations at the L, N, and S sites (https://www.timeanddate.com/weather/poland/torun/historic?month=7&year=2022, accessed on 4 February 2023) [45] (Table 2). Nitrification increased over time at G8 (Table S7). NH_4_^+^ increased at the sandy S site but declined at the G site, consistent with the drought effects on microbial nitrification [46]. Under drought stress, sandy soil may alter the nitrogen-cycling pathways via microbial community shifts [47,48] (Table 2).

Soil P cycling and bioavailability are closely related to water dynamics [49]. Drought reduces enzyme activity and shifts P from inorganic to organic forms [49,50]. Sites G, L, and N showed lower total P levels, while sites L and N showed lower total and available P levels at T8 (*p* < 0.05) (Table 2). Although calcareous soils frequently immobilize P, the high correlation between total P and available P (r = 1.00 in G8) indicates that microbial phosphatases mediate rapid P cycling [51]. This suggests that short-term organic matter inputs (such as root exudates) may momentarily increase P availability, which contrasts with the usual P fixation in high-pH soils. Moreover, very few studies are conducted to study the relationship between soil pH and phosphorus contents. Sites G and S showed higher pH and available P under drought conditions (Table 2), aligning with studies showing increased P levels released at pH 6.0–7.1 [52]. Furthermore, soils with slightly alkaline and calcific properties (Table 2, sites S and G) tend to retain P by precipitating insoluble Ca-P species [53,54]. These results highlight the indirect effect of drought on nutrient availability, which is crucial for climate change assessments.

### 4.2. Impact of Soil Moisture on Culturable Microbial Diversity

Bacterial abundance exceeded that of actinomycetes and fungi, remaining stable under low soil moisture, which is consistent with [55] and supported by our data (Table S3 and Figure 4). Microbial abundance is influenced by soil moisture availability and pore heterogeneity [5,9,56]. Actinomycetes contributed to phosphorus release, particularly at the humus-enriched G site (Table S7). Bacteria correlated with nitrate levels (r = 1.00 at G0, L8, N8, S8), reflecting nitrification and urease activity (Table S7), even in sandy soils, where nitrate is prone to leaching. These challenges studies showing nitrate depletion in drought-stressed soils [57]. Despite drought stress, nitrate was sustained by organic N mineralization and microbial activity [23,57]. Our results show dominance of bacterial communities even in nutrient-poor sandy soils, contrary to studies such as [58], which demonstrated bacterial dominance in organic-rich soils.

Contrary to many studies showing fungal increase under drought, fungal abundance declined with decreasing moisture (Table S3 and Figure 4) and correlated weakly with moisture (r = 0.76–0.83) [1,16,19,59]. The fungi in sandy soils appear moisture-dependent, likely due to nutrient limitations and pore size effects protecting bacteria better under dry conditions [60,61,62]. Bacteria may survive in minimal water in small pores or biofilms, maintaining habitats under drought conditions [61,62]. Our findings highlight the need to investigate microbial dynamics across microenvironments under moisture fluctuations [63,64].

The PCA revealed site-specific microbial partitioning: actinomycetes at G, bacteria and fungi at L, and mixed communities at N and S, reflecting rapid (<2 months) functional divergence, possibly linked to fertilization history [65]. In this study, bacteria dominate nitrogen cycling, actinomycetes specialize in phosphorus release, and fungi thrive in moist environments, contributing to organic matter decomposition.

### 4.3. Impact of Soil Moisture on Enzyme Activities

Soil enzymes act as indicators of soil health, integrating physical, chemical and microbial conditions. They are mainly produced by bacteria and fungi, influencing soil fertility and nutrient availability [1]. In our study, microbial abundance correlated positively with enzyme activities at T0 and T8 (Table S5), highlighting their biological importance.

A sharp reduction in soil moisture (88.74–96.91%) led to the significant changes in four key enzymes (Table S4 and Figure 5). Soil moisture strongly influences enzyme activity, particularly alkaline phosphatase (AKP), suggesting enhanced phosphorus cycling in wetter soils. Urease activity showed weak correlation at G8, possibly due to saturation or anaerobic conditions (Table S7). Dehydrogenase (DH), key to carbon cycling, declined sharply under drought conditions (except for the L site), showing a rapid microbial metabolic shutdown (Table S4 and Figure 6), especially in sandy soil (S site). This aligns with studies showing DH is highly sensitive to moisture [1,8].

In addition, the drought-induced impact was more pronounced for ACP, which was lower compared to AKP (Table S4 and Figure 6), and these results agree with the soil pH range (between 6 and 8.3), i.e., closer to the alkaline pH (Table 2). While drought typically reduces both AKP and ACP [18,66], we observed high AKP resilience, possibly due to the buffering effects of CaCO_3_ or microbial adaptations (Table S7). At T8, AKP dominated total phosphatase activity (93.49%), while ACP activity, highest at the nutrient-poor S site, declined. This suggests drought-tolerant microbes (e.g., *Bacillus*) promote ACP activity even in dry sandy soil.

Urease (UR) activity increased under drought conditions, particularly at the G site (Figure 6), correlating with nitrate (r = 0.88–0.98). Despite moisture loss, this suggests microbial shifts (e.g., Actinobacteria dominance at the N site) or compensatory nitrogen mineralization. Initially, the low UR activity at the G and N sites may be due to high ammonium levels, which inhibit urease [44]. At the S site, lower ammonium and microbial biomass led to increased UR, indicating nitrogen demand [1,67,68].

The PCA analysis revealed site-specific enzymatic patterns: G/N/S sites showed distinct enzyme profiles, while L remained inactive. This rapid functional divergence within weeks reflects management impacts and reveals enzyme-based niche separation, beyond traditional soil classifications [69].

### 4.4. Carbon Substrate Utilization Patterns Based on Community-Level Physiology Profiling (BIOLOG-CLPPs)

BIOLOG plate color development reflects bacterial activity in response to carbon sources and helps compare microbial communities [5,19]. The metabolic diversity of the soil community was higher at T0 at all sites compared to T8 (Figure S3 and Figure 7). This drop may result from microbial substrate preferences or drought-induced stress. Some carbon sources (e.g., 4-hydroxy benzoic acid, α-ketobutyric acid, L-phenylalanine, and α-cyclodextrin) remain unaffected, suggesting drought treatment may not be severe enough (in terms of duration or water loss) to cause meaningful alterations in soil functional diversity. Despite reduced moisture, the G site maintained high metabolic activity (carbohydrates, amino acids), while the L site used few carboxylic acids/amines, and the S site preserved richness. Carbon and amino acids showed strong correlation with NH_4_^+^ levels (r = 1.00 across all sites/time points), indicating that nitrogen plays a key role in microbial metabolism under drought conditions. Contrary to expectations, the S site sustained microbial richness despite low organic carbon, challenging assumptions that sandy soil rapidly loses diversity [56].

Polymers were strongly associated with CaCO_3_ (r = 1.00 in G8/S8), suggesting that calcareous soils may support polymer-degrading microbes such as Actinobacteria, even under drought conditions [70]. Carbohydrate metabolism also remained strongly linked to organic carbon (r = 1.00 in G0/S0), even in the low-OC sandy soil. While richness declined sharply in L and G, evenness remained stable, pointing to dominance by stress-tolerant taxa such as Proteobacteria [56,70]. Our observed rapid functional shifts (<2 months) challenge views that diversity changes require long-term management [12]. In general, drought reduced microbial activity and diversity, especially in nutrient-rich soils, while sandy soil showed unexpected resilience. Carboxylic acids reflect organic matter breakdown, carbohydrates support energy metabolism, polymers correlate with CaCO_3_ stabilization, and amino acids align with nitrogen mineralization across the L, N and S sites (Table S7).

### 4.5. Impact of Drought Stress on Total Microbial Communities (16S and ITS)

The variations in the relative abundance of microbial taxa under drought conditions differed notably between sites and appear to be shaped by local environmental factors such as soil pH, organic matter content, and moisture availability. Acidobacteriota increased at the G site (6.35% → 8.77%) but significantly decreased at the L site (26.23% → 7.93%) (Figure 9), likely due to differences in nutrient availability and soil buffering capacity [64,71,72]. As these bacteria thrive under stable moisture conditions and low-nutrient environments, their decline in L indicates sensitivity to drought, while the increase at the G site suggests that organic matter helped maintain suitable conditions. Although Actinobacteria are common in dry soils, their decline at the G and N sites under increasing drought stress implies that even drought-resistant taxa are affected by severe moisture loss, possibly due to reduced organic carbon or pH changes [72]. Bacteroidota, which are known degraders of organic matter, increased at the S site, suggesting a shift toward bacteria adapted to rapidly changing conditions [64,71]. Firmicutes increased at the L and S sites, likely due to their ability to form endospores and perform anaerobic metabolism [71,73]. In contrast, Proteobacteria declined under drought conditions, especially at the L site, pointing to reduced fixation because of their desiccation sensitivity [1]. The less significant results are indicated in the Supplementary File (Discussion, Section S4.5).

Several functionally important taxa also responded distinctly. *Blautia*, a carbon-associated anaerobe, declined under drought conditions, indicating reduced labile carbon availability [74]. *Bradyrhizobium* remained comparatively stable (Figure 9) due to its ability to fix nitrogen even under low soil moisture conditions [75,76]. *Bryobacter* and *Eisenbergiella* declined, reflecting sensitivity to drought-induced changes in carbon and anaerobic niches [77,78]. Meanwhile, the cyanobacterium *Hassallia* increased at the S site, suggesting enhanced drought resilience through moisture retention and nitrogen fixation [79]. The rise in *Nitrospira* indicates a shift toward bacterial nitrification in response to reduced nitrogen fixation [80]. While the Shannon index slightly decreased at the S site (7.06 → 7.03), it increased at the G, L, and N sites, likely due to niche partitioning or past organic inputs. The less significant results are indicated in the Supplementary File (Discussion, Section S4.5). The Simpson evenness remained high across all sites, indicating functional redundancy despite changes in dominant taxa (e.g., Actinobacteria). The greatest disparity in Chao1 richness at G0 reflects a potential underestimation of rare taxa in organic-rich soils.

In the fungal community, Ascomycota remained dominant (48.98% to 81.67%) but declined over time in most sites, especially at the G (13%), L (16.25%), and S (4.76%) sites, indicating drought sensitivity, contrary to some studies [63,81]. These site-specific declines may result from differences in management or soil type. In contrast, their increase at the N site (48.98% to 68.60%) may reflect drought-tolerant genera such as *Trichoderma* and *Chaetomium* [82,83]. The less-significant results are indicated in the Supplementary File (Discussion, Section S4.5). Basidiomycota showed strong enrichment at N0 (Figure 9) but significantly decreased at the N site (41.19% to 9.44%), indicating that they are extremely susceptible to soil moisture loss and dependent on stable soil conditions [84]. Mortierellomycota increased at the G, L, and N sites, suggesting the presence of opportunistic or drought-tolerant species capable of surviving under low-nutrient and stressful situations [32,85,86]. Nonetheless, a drop in S (4.42%) indicates localized soil moisture limitations. This contrasts with studies linking Mortierellomycota to drought resilience [32,85,86].

Some fungal genera responded more distinctly. *Fusarium* increased at the L and S sites, potentially benefiting from fluctuating moisture in mixed-texture soils [87,88]. *Gibellulopsis* and *Fusicolla* increased at the L site, suggesting its ability to survive in transitional clay–sandy soils [10,89,90]. The xerotolerant fungi such as *Cladosporium* and *Exophiala* persisted or increased in abundance, reflecting drought adaptation [91]. The *Solicoccozyma*, tolerant to dry and nutrient-poor conditions, was able to survive in sandy soils such as S in [92,93]. *Hymenoscyphus* increased at the N site, likely due to the moisture-retaining nature of clay [94]. Meanwhile *Panaeolina* declined at all sites, especially in compacted N soil, supporting the idea that basidiomycetes are particularly sensitive to low moisture [84,95]. The less-significant results are indicated in the Supplementary File (Discussion, Section S4.5). The overall decline in the fungal relative abundance in S may be tied to poor water retention in sandy soils [96].

The fungal diversity, as measured by the Shannon Index, decreased notably at the S (5.27 → 3.90) but increased at the G, L, and N sites, consistent with better moisture retention and greater niche diversity in clay-rich soils [81]. The drop in evenness at the S site (0.99 → 0.95) contrasts with the increase at other sites (0.96 → 0.99), highlighting how drought stress leads to dominance by a few taxa in sandy soils, whereas clay soils maintain more balanced communities. These findings support the idea that drought reduces microbial diversity and drives community shifts towards stress-tolerant groups. Understanding these dynamics is key to managing soils under increasing drought conditions.

## 5. Conclusions

Drought conditions are an ongoing threat in the world, especially in agricultural land. Soil-water content has a fundamental role in understanding the soil biological activities because in this study, water deficit conditions were clearly correlated with soil microbes, their enzymes and functional diversity. In general, the data shows notable changes over time in the investigated soil physicochemical as well as biological parameters. Increased phosphorus availability, fungal activity, and organic matter stabilization were observed at T8; however, bacterial activity and rapid nitrogen cycling predominated overall at T0. A key factor influencing microbial activity and nutrient availability is soil moisture. There is a strong correlation between carbon and nitrogen, as well as biological substances such as amino acids and carbohydrates. Actinomycetes support enzymatic activity that increases phosphorus cycling. Calcium carbonate stabilizes polymers during breakdown and buffers the pH. Since there is little calcium carbonate present, pH affects enzyme activity in the L, N, and S sites but stays constant over time. This pattern suggests that soil moisture plays a major role in soil biological activities, raising new questions about the potential role of climate change, especially drought conditions, on soil microbial communities, their enzymes, and functional diversity. Our results provide evidence that as little as 2 months of drought can alter soil microbial communities in agricultural lands. Large gaps of information remain for the effect of drought on agricultural soils. Meta-analysis or combination of advanced molecular techniques may be taken into consideration in future for better identification of changes in soil biological activities in response to drought conditions.

## Figures and Tables

**Table 1:** Soil grain fraction percentage at four investigated sites (G: Gniewkowo, L: Lulkowo, N: Nieszawa, and S: Suchatówka).

Sites	Clay Content (<0.002 mm, %)		Sand (2–0.05 mm, %)		Silt (0.05–0.002 mm, %)	
	T0	T8	T0	T8	T0	T8
G	4	5	86	83	10	12
L	3	4	88	85	9	11
N	8	8	72	70	20	22
S	5	2	84	91	11	7

T0; sampling date, T8; 8th week.

**Table 2:** Soil chemical properties of four investigated soil samples at zero sampling day (T0) and 8th week (T8) of induced drought conditions (G: Gniewkowo; L: Lulkowo; N: Nieszawa, S: Suchatówka; C: organic carbon; CaCO_3_: calcium carbonate; N: total nitrogen; NO_3_^−^: nitrate; NH_4_^+^: ammonium; P: total phosphorus; P_2_O_5_: available phosphorus, *; *p* < 0.05).

Location	C [%]		N [%]		P [mg/kg]		P_2_O_5_ [mg/kg]		NO_3_^−^		NH_4_^+^ [mg/kg]		pH		CaCO_3_ [%]	
									[mg/kg]							
	T0	T8	T0	T8	T0	T8	T0	T8	T0	T8	T0	T8	T0	T8	T0	T8
G	1.22 ± 0.025	1.16 ± 0.005	0.117 ± 0.003	0.124 ± 0.002	434 ± 1.87	363 ± 2.5 *	25.2 ± 0.34	116.2 ± 0.35 *	58.2 ± 1.10	93.7 ± 0.51 *	2.26 ± 0.13	1.8 ± 0.10 *	8.2 ± 0.25	8.3 ± 0.32	1.6 ± 0.15	1.6 ± 0.06
L	0.71 ± 0.106	0.72 ± 0.007	0.074 ± 0.001	0.076 ± 0.001	460 ± 2.00	432 ± 2.5 *	119 ± 1.00	32.4 ± 0.31 *	30.5 ± 0.25	57.3 ± 0.25 *	1.02 ± 0.03	1.02 ± 0.01	6 ± 0.12	6.1 ± 0.06	0 ± 0.00	0 ± 0.00
N	1.37 ± 0.005	1.27 ± 0.006 *	0.163 ± 0.001	0.155 ± 0.001 *	488 ± 2.5	436 ± 0.99 *	38.4 ± 0.36	30.4 ± 0.31 *	446.7 ± 0.15	345.2 ± 0.25 *	0.79 ± 0.02	0.64 ± 0.02	7.1 ± 0.10	7.2 ± 0.15	0.54 ± 0.02	0.54 ± 0.01
S	0.76 ± 0.003	0.67 ± 0.007	0.066 ± 0.001	0.059 ± 0.002 *	454 ± 1.63	468 ± 1.91 *	22.8 ± 0.15	32.7 ± 0.25 *	30.6 ± 0.26	39.4 ± 0.31 *	0.57 ± 0.02	0.64 ± 0.03 *	8.2 ± 0.15	8.2 ± 0.15	3.8 ± 0.15	3.2 * ± 0.06

**Table 3:** Functional diversity index analysis from Community Level Physiological Profiles (CLPPs)—Shannon, richness and evenness. G: Gniewkowo, L: Lulkowo, N: Wielka Nieszawka, S: Suchatówka, 0: collection date, 8: 8 weeks of drought.

Functional Diversity INDEX	G0	G8	L0	L8	N0	N8	S0	S8
1. Shannon–Wiener index (H)	3.34	2.93	3.3	2.97	3.26	3.03	3.17	3
2. Richness	29	20	28	15	26	19	25	17
3. Evenness	0.972	0.853	0.96	0.865	0.949	0.884	0.924	0.875

**Table 4:** Relative abundance of 16S amplicon sequence. G: Gniewkowo, L: Lulkowo, N: Wielka Nieszawka, S: Suchatówka, 0: collection date, 8: 8 weeks of drought.

		Relative Abundance (%)							
		G0	G8	L0	L8	N0	N8	S0	S8
A. Phyla	Acidobacteriota	6.35	8.77	26.23	7.93	11.42	10.59	17.69	12.17
	Actinobacteriota	27.70	22.77	7.98	12.05	19.49	16.42	15.75	12.26
	Bacteroidota	19.40	18.31	13.15	19.58	19.92	19.15	16.81	21.36
	Crenarchaeota	0.14	1.04	1.04	0.33	1.31	0.33	2.09	4.23
	Firmicutes	13.43	12.44	13.00	21.01	8.79	10.18	7.80	11.95
	Gemmatimonadota	1.86	1.83	2.80	2.84	0.67	2.91	0.54	1.98
	Nitrospirota	0.93	2.68	0.62	3.47	2.53	3.10	2.09	4.23
	Proteobacteria	16.95	15.67	27.18	17.58	19.50	16.78	18.44	16.24
B. Genera	*Blautia*	1.52	0.75	1.13	1.05	0.41	0.64	0.77	0.97
	*Bradyrhizobium*	0.24	0.23	0.31	0.33	0.36	0.26	0.28	0.38
	*Bryobacter*	0.06	0.05	2.04	0.08	0.04	0.05	0.04	0.04
	*Eisenbergiella*	0.52	0.21	0.43	0.20	0.19	0.14	0.21	0.41
	*Ellin6055*	1.20	0.63	0.17	0.51	1.11	0.74	1.14	0.44
	*Hassallia*	0.29	0.38	0.00	0.37	0.28	0.32	0.45	0.95
	*Nitrospira*	0.26	0.72	0.08	0.54	0.46	0.62	0.31	1.10
	*Parabacteroides*	1.06	0.47	0.77	0.53	0.24	0.30	0.34	0.64
	*Pseudarthrobacter*	2.49	1.09	0.16	0.36	0.45	0.40	0.62	0.69
	*Pseudolabrys*	0.09	0.06	2.62	0.08	0.00	0.06	0.06	0.06
	*RB41*	0.26	0.34	0.12	0.25	0.32	0.42	0.64	1.17

**Table 5:** Bacterial alpha diversity indices: Shannon and Simpson indices. G: Gniewkowo, L: Lulkowo, N: Wielka Nieszawka, S: Suchatówka, 0: sampling day, 8: 8 weeks of drought.

Index	G0	G8	L0	L8	N0	N8	S0	S8
Shannon	7.13	7.21	6.33	7.04	7.09	7.23	7.06	7.03
Simpson	1.00	1.00	0.99	1.00	1.00	1.00	1.00	1.00
Observed	4063.00	3100.00	2006.00	2837.00	2942.00	3153.00	2934.00	2875.00
Chao1	5446.29	3137.56	2007.88	2856.80	2983.28	3197.27	2967.62	2908.39
se.chao1	113.09	8.84	1.54	5.76	9.30	9.65	8.13	8.23

**Table 6:** Relative abundance of ITS amplicon sequence. G: Gniewkowo, L: Lulkowo, N: Wielka Nieszawka, S: Suchatówka, 0: sampling day, 8: 8 weeks of drought.

		Relative Abundance (%)							
		G0	G8	L0	L8	N0	N8	S0	S8
A. Phyla	Ascomycota	79.75	66.75	81.67	65.42	48.98	68.60	70.06	65.30
	Basidiomycota	8.93	8.13	12.23	11.11	41.19	9.44	7.34	4.91
	Mortierellomycota	4.46	6.05	0.27	9.68	5.07	6.74	5.78	1.37
	Chytridiomycota	0.38	1.01	0.21	4.36	0.54	3.33	0.57	0.55
B. Genera	*Cladosporium*	1.93	1.18	0.03	1.73	3.20	3.15	3.70	1.89
	*Exophiala*	0.18	0.79	0.05	0.41	0.69	0.66	2.14	1.65
	*Fusarium*	5.93	0.78	0.00	2.90	0.82	0.49	3.04	6.10
	*Fusicolla*	3.28	3.13	0.00	3.12	1.70	0.96	2.39	0.56
	*Gibellulopsis*	4.66	2.29	0.07	2.79	1.12	0.68	0.24	0.05
	*Hymenoscyphus*	0.02	0.00	0.00	0.00	0.02	17.10	0.03	0.03
	*Panaeolina*	0.04	0.00	0.00	0.00	33.80	0.06	0.05	0.04
	*Plectosphaerella*	25.02	3.69	0.00	0.59	0.17	1.05	0.23	0.15
	*Ramophialophora*	0.09	0.00	0.00	0.21	0.04	0.59	0.00	7.21
	*Solicoccozyma*	1.46	1.01	0.04	0.25	0.43	0.24	2.16	0.36
	*Trichoderma*	0.00	0.00	0.00	6.32	0.00	1.37	0.13	0.03

**Table 7:** Fungal alpha diversity indices: Shannon and Simpson indices. G: Gniewkowo, L: Lulkowo, N: Wielka Nieszawka, S: Suchatówka, 0: sampling day, 8: 8 weeks of drought.

	G0	G8	L0	L8	N0	N8	S0	S8
Shannon	4.19	5.35	3.95	5.02	4.2	4.89	5.27	3.9
Simpson	0.96	0.99	0.97	0.98	0.88	0.96	0.99	0.95
Observed	548	561	277	684	832	884	905	480
Chao1	548	561	277	684	832	884	905	480
se.chao1	0.01	0	0	0	0	0.01	0	0

## Data Availability

The raw data supporting the conclusion of this manuscript will be made available by the authors, without undue reservation, to any qualified researcher. DNA sequencing data is available at NCBI SRA BioProject ID PRJNA1226519.

## Supplementary Materials

The following supporting information can be downloaded at: https://www.mdpi.com/article/10.3390/microorganisms13061245/s1, Table S1. Media composition (g/L); Table S2. Pearson correlation matrix with respective r values between soil moisture and physicochemical at zero sampling day (T0) and 8th week (T8) of induced drought conditions; Table S3. Enumeration of bacteria, fungi and actinomycetes in four agricultural soils; Table S4. Pearson correlation matrix with respective r values between soil moisture and biological parameters at 0, 1, 2, 4, and 8 weeks of induced drought conditions; Table S5. Pearson correlation matrix with respective r values between microbial abundance and enzymatic activities at T0 and T8 weeks under induced drought conditions; Table S6. Pearson correlation matrix with respective r values between soil moisture and major carbon sources; Table S7. Pearson correlation matrix with respective r values between soil physicochemical and biological factors at zero sampling day (T0) and 8th week (T8) of induced drought conditions; Figure S1. Principal component analysis (PCA) of (A) the studied correlations between soil moisture content and physicochemical parameters at T0 and T8, (B) the respective contribution of each component to their total variability. G, Gniewkowo; L, Lulkowo; N, Wielka Nieszawa; S, Suchatówka; 0, week 0; 8, week 8; Figure S2. Principal component analysis (PCA) indicating correlations between soil moisture content and biological parameters in four soil samples at investigated time intervals; Figure S3. Absorbance values of Biolog-Ecoplates in four types of agricultural soil samples with carbon substrate utilization efficiency.

## References

[1] Bogati K., Walczak M. (2022). The Impact of Drought Stress on Soil Microbial Community, Enzyme Activities and Plants. Agronomy.

[2] Ghimire S., Deo R.C., Downs N.J., Raj N. (2019). Global Solar Radiation Prediction by ANN Integrated with European Centre for Medium Range Weather Forecast Fields in Solar Rich Cities of Queensland Australia. J. Clean. Prod..

[3] Danilovich I.S., Loginov V.F., Groisman P.Y. (2023). Changes of Hydrological Extremes in the Center of Eastern Europe and Their Plausible Causes. Water.

[4] Orimoloye I.R. (2022). Agricultural Drought and Its Potential Impacts: Enabling Decision-Support for Food Security in Vulnerable Regions. Front. Sustain. Food Syst..

[5] Preece C., Farré-Armengol G., Peñuelas J. (2020). Drought Is a Stronger Driver of Soil Respiration and Microbial Communities than Nitrogen or Phosphorus Addition in Two Mediterranean Tree Species. Sci. Total Environ..

[6] Liu H., Ren F., Wan S., Han S., Zheng J. (2025). Nitrogen and Water Additions with or without Mowing Altered Soil Microbial Community Characteristics in a Semi-Arid Steppe. Ecol. Process..

[7] Singh S., Mayes M.A., Shekoofa A., Kivlin S.N., Bansal S., Jagadamma S. (2021). Soil Organic Carbon Cycling in Response to Simulated Soil Moisture Variation under Field Conditions. Sci. Rep..

[8] Gomez E.J., Delgado J.A., Gonzalez J.M. (2021). Influence of Water Availability and Temperature on Estimates of Microbial Extracellular Enzyme Activity. PeerJ.

[9] Schimel J.P. (2018). Life in Dry Soils: Effects of Drought on Soil Microbial Communities and Processes. Annu. Rev. Ecol. Evol. Syst..

[10] Zhang H., Dong L., Yao X., Wang W. (2023). Soil Fertility Shifts the Relative Importance of Saprotrophic and Mycorrhizal Fungi for Maintaining Ecosystem Stability. Glob. Change Biol..

[11] Wolf S., Paul-Limoges E. (2023). Drought and Heat Reduce Forest Carbon Uptake. Nat. Commun..

[12] Leyrer V., Blum J., Marhan S., Kandeler E., Zimmermann T., Berauer B.J., Schweiger A.H., Canarini A., Richter A., Poll C. (2025). Drought Impacts on Plant–Soil Carbon Allocation—Integrating Future Mean Climatic Conditions. Glob. Change Biol..

[13] Moreno-Gámez S., Kiviet D.J., Vulin C., Schlegel S., Schlegel K., Van Doorn G.S., Ackermann M. (2020). Wide Lag Time Distributions Break a Trade-off between Reproduction and Survival in Bacteria. Proc. Natl. Acad. Sci. USA.

[14] Najera F., Dippold M.A., Boy J., Seguel O., Koester M., Stock S., Merino C., Kuzyakov Y., Matus F. (2020). Effects of Drying/Rewetting on Soil Aggregate Dynamics and Implications for Organic Matter Turnover. Biol. Fertil. Soils.

[15] Bogati K.A., Golińska P., Sewerniak P., Burkowska-But A., Walczak M. (2023). Deciphering the Impact of Induced Drought in Agriculture Soils: Changes in Microbial Community Structure, Enzymatic and Metabolic Diversity. Agronomy.

[16] Hestrin R., Kan M., Lafler M., Wollard J., Kimbrel J.A., Ray P., Blazewicz S.J., Stuart R., Craven K., Firestone M. (2022). Plant-Associated Fungi Support Bacterial Resilience Following Water Limitation. ISME J..

[17] Semenov M.V., Zhelezova A.D., Ksenofontova N.A., Ivanova E.A., Nikitin D.A., Semenov V.M. (2025). Microbiological Indicators for Assessing the Effects of Agricultural Practices on Soil Health: A Review. Agronomy.

[18] Qu Q., Wang Z., Gan Q., Liu R., Xu H. (2023). Impact of Drought on Soil Microbial Biomass and Extracellular Enzyme Activity. Front. Plant Sci..

[19] Bogati K., Sewerniak P., Walczak M. (2023). Effect of Changes in Soil Moisture on Agriculture Soils: Response of Microbial Community, Enzymatic and Physiological Diversity. Ecol. Quest..

[20] Grzyb A., Wolna-Maruwka A., Łukowiak R., Ceglarek J. (2022). Spatial and Temporal Variability of the Microbiological and Chemical Properties of Soils under Wheat and Oilseed Rape Cultivation. Agronomy.

[21] Jia X., Zhong Y., Liu J., Zhu G., Shangguan Z., Yan W. (2020). Effects of Nitrogen Enrichment on Soil Microbial Characteristics: From Biomass to Enzyme Activities. Geoderma.

[22] Koner S., Chen J.-S., Hsu B.-M., Tan C.-W., Fan C.-W., Chen T.-H., Hussain B., Nagarajan V. (2021). Assessment of Carbon Substrate Catabolism Pattern and Functional Metabolic Pathway for Microbiota of Limestone Caves. Microorganisms.

[23] Siebielec S., Siebielec G., Klimkowicz-Pawlas A., Gałązka A., Grządziel J., Stuczyński T. (2020). Impact of Water Stress on Microbial Community and Activity in Sandy and Loamy Soils. Agronomy.

[24] Yang J., Wang J., Li A., Li G., Zhang F. (2020). Disturbance, Carbon Physicochemical Structure, and Soil Microenvironment Codetermine Soil Organic Carbon Stability in Oilfields. Environ. Int..

[25] Naár Z. (2006). Redundancy Analysis of the Influence of Metal Content and other Edaphic Parameters on the Co- Exsistence of Trichoderma Species. Appl. Ecol. Environ. Res..

[26] Rong L., Duan X., Feng D., Zhang G. (2017). Soil Moisture Variation in a Farmed Dry-Hot Valley Catchment Evaluated by a Redundancy Analysis Approach. Water.

[27] Xiao Y.S., Zhou B., Han Z., Liu S., Ding C., Jia F., Zeng W. (2022). Microbial Mechanism of Zinc Fertilizer Input on Rice Grain Yield and Zinc Content of Polished Rice. Front. Plant Sci..

[28] Davoudabadi M.J., Pagendam D., Drovandi C., Baldock J., White G. (2024). Innovative Approaches in Soil Carbon Sequestration Modelling for Better Prediction with Limited Data. Sci. Rep..

[29] Pierson D., Lohse K.A., Wieder W.R., Patton N.R., Facer J., De Graaff M.-A., Georgiou K., Seyfried M.S., Flerchinger G., Will R. (2022). Optimizing Process-Based Models to Predict Current and Future Soil Organic Carbon Stocks at High-Resolution. Sci. Rep..

[30] Capblancq T., Forester B.R. (2021). Redundancy Analysis: A Swiss Army Knife for Landscape Genomics. Methods Ecol. Evol..

[31] Chen H., Ma K., Lu C., Fu Q., Qiu Y., Zhao J., Huang Y., Yang Y., Schadt C.W., Chen H. (2022). Functional Redundancy in Soil Microbial Community Based on Metagenomics Across the Globe. Front. Microbiol..

[32] Liu Y., Men M., Peng Z., Houx J.H., Peng Y. (2022). Nitrogen Availability Determines Ecosystem Productivity in Response to Climate Warming. Ecology.

[33] Gebhardt H. (1982). Phosphatkartierung Und Boden Kundliche Geländeuntersuchungen Zur Eingrenzung Historischer Siedlungs-Und Wirtschaftsflächen Der Geestinsel Flogeln. Probleme der Küstenforschung im Südlichen Nordseegebiet. Hildesheim.

[34] Van Reeuwijk L.P. (2002). Procedures for Soil Analysis.

[35] Bednarek R., Dziadowiec H., Pokojska U., Prusinkiewicz Z. (2005). Eco-Pedological Studies.

[36] Warzyński H., Sosnowska A., Harasimiuk A. (2018). Effect of Variable Content of Organic Matter and Carbonates on Results of Determination of Granulometric Composition by Means of Casagrande’s Areometric Method in Modification by Prószyński. Soil. Sci. Annu..

[37] Furtak K., Grządziel J., Gałązka A., Niedźwiecki J. (2019). Analysis of Soil Properties, Bacterial Community Composition, and Metabolic Diversity in Fluvisols of a Floodplain Area. Sustainability.

[38] Kandeler E., Gerber H. (1988). Short-Term Assay of Soil Urease Activity Using Colorimetric Determination of Ammonium. Biol. Fertil. Soils.

[39] Dumelle M., Higham M., Ver Hoef J.M. (2023). Spmodel: Spatial Statistical Modeling and Prediction in R. PLoS ONE.

[40] Xia Y., Sun J., Chen D.-G. (2018). Statistical Analysis of Microbiome Data with R.

[41] Wen T., Niu G., Chen T., Shen Q., Yuan J., Liu Y.-X. (2023). The Best Practice for Microbiome Analysis Using R. Protein Cell.

[42] Zhong Z., Chen Z., Xu Y., Ren C., Yang G., Han X., Ren G., Feng Y. (2018). Relationship between Soil Organic Carbon Stocks and Clay Content under Different Climatic Conditions in Central China. Forests.

[43] Ayari M., Charef A., Azouzi R., Trifi M., Khiari N. (2023). Impact of Induced Natural Organic Carbons on Soil Organic Carbon (SOC), Permeability and Production of Sandy and Clay Soils in Mediterranean Semi-Arid Eco-System. Commun. Soil. Sci. Plant Anal..

[44] Deng L., Peng C., Kim D.-G., Li J., Liu Y., Hai X., Liu Q., Huang C., Shangguan Z., Kuzyakov Y. (2021). Drought Effects on Soil Carbon and Nitrogen Dynamics in Global Natural Ecosystems. Earth-Sci. Rev..

[45] Gao W., Reed S.C., Munson S.M., Rui Y., Fan W., Zheng Z., Li L., Che R., Xue K., Du J. (2021). Responses of Soil Extracellular Enzyme Activities and Bacterial Community Composition to Seasonal Stages of Drought in a Semiarid Grassland. Geoderma.

[46] Silva I., Alves M., Malheiro C., Silva A.R.R., Loureiro S., Henriques I., González-Alcaraz M.N. (2022). Short-Term Responses of Soil Microbial Communities to Changes in Air Temperature, Soil Moisture and UV Radiation. Genes.

[47] Parker S.S., Schimel J.P. (2011). Soil Nitrogen Availability and Transformations Differ between the Summer and the Growing Season in a California Grassland. Appl. Soil. Ecol..

[48] Yue P., Zuo X., Li K., Cui X., Wang S., Misselbrook T., Liu X. (2021). The Driving Effect of Nitrogen-Related Functional Microorganisms under Water and Nitrogen Addition on N_2_O Emission in a Temperate Desert. Sci. Total Environ..

[49] Hinojosa M.B., Laudicina V.A., Parra A., Albert-Belda E., Moreno J.M. (2019). Drought and Its Legacy Modulate the Post-fire Recovery of Soil Functionality and Microbial Community Structure in a Mediterranean Shrubland. Glob. Change Biol..

[50] Margalef O., Sardans J., Maspons J., Molowny-Horas R., Fernández-Martínez M., Janssens I.A., Richter A., Ciais P., Obersteiner M., Peñuelas J. (2021). The Effect of Global Change on Soil Phosphatase Activity. Glob. Change Biol..

[51] Chamberlain L.A., Aguayo T., Zerega N.J.C., Dybzinski R., Egerton-Warburton L.M. (2022). Rapid Improvement in Soil Health Following the Conversion of Abandoned Farm Fields to Annual or Perennial Agroecosystems. Front. Sustain. Food Syst..

[52] Sato S., Comerford N.B. (2005). Influence of Soil pH on Inorganic Phosphorus Sorption and Desorption in a Humid Brazilian Ultisol. Rev. Bras. Ciênc. Solo.

[53] Wu L., Zhang S., Wang J., Ding X. (2020). Phosphorus Retention Using Iron (II/III) Modified Biochar in Saline-Alkaline Soils: Adsorption, Column and Field Tests. Environ. Pollut..

[54] Audette Y., Smith D.S., Parsons C.T., Chen W., Rezanezhad F., Van Cappellen P. (2020). Phosphorus Binding to Soil Organic Matter via Ternary Complexes with Calcium. Chemosphere.

[55] Borowik A., Wyszkowska J. (2016). Soil Moisture as a Factor Affecting the Microbiological and Biochemical Activity of Soil. Plant Soil. Environ..

[56] Malik A.A., Bouskill N.J. (2022). Drought Impacts on Microbial Trait Distribution and Feedback to Soil Carbon Cycling. Funct. Ecol..

[57] Fink J.W., Held N.A., Manhart M. (2023). Microbial Population Dynamics Decouple Growth Response from Environmental Nutrient Concentration. Proc. Natl. Acad. Sci. USA.

[58] Koch B.J., McHugh T.A., Hayer M., Schwartz E., Blazewicz S.J., Dijkstra P., Van Gestel N., Marks J.C., Mau R.L., Morrissey E.M. (2018). Estimating Taxon-specific Population Dynamics in Diverse Microbial Communities. Ecosphere.

[59] Frey B., Walthert L., Perez-Mon C., Stierli B., Köchli R., Dharmarajah A., Brunner I. (2021). Deep Soil Layers of Drought-Exposed Forests Harbor Poorly Known Bacterial and Fungal Communities. Front. Microbiol..

[60] Wagg C., Dudenhöffer J., Widmer F., Van Der Heijden M.G.A. (2018). Linking Diversity, Synchrony and Stability in Soil Microbial Communities. Funct. Ecol..

[61] Benucci G.M.N., Toosi E.R., Yang F., Marsh T.L., Bonito G.M., Kravchenko A. (2023). The Microbiome Structure of Decomposing Plant Leaves in Soil Depends on Plant Species, Soil Pore Sizes, and Soil Moisture Content. Front. Microbiol..

[62] Navas M., Martín-Lammerding D., Hontoria C., Ulcuango K., Mariscal-Sancho I. (2021). The Distinct Responses of Bacteria and Fungi in Different-sized Soil Aggregates under Different Management Practices. Eur. J. Soil. Sci..

[63] Jaeger A.C.H., Hartmann M., Conz R.F., Six J., Solly E.F. (2024). Prolonged Water Limitation Shifts the Soil Microbiome from Copiotrophic to Oligotrophic Lifestyles in Scots Pine Mesocosms. Environ. Microbiol. Rep..

[64] Wan Q., Li L., Liu B., Zhang Z., Liu Y., Xie M. (2023). Different and Unified Responses of Soil Bacterial and Fungal Community Composition and Predicted Functional Potential to 3 Years’ Drought Stress in a Semiarid Alpine Grassland. Front. Microbiol..

[65] Xu Q., Li L., Guo J., Guo H., Liu M., Guo S., Kuzyakov Y., Ling N., Shen Q. (2024). Active Microbial Population Dynamics and Life Strategies Drive the Enhanced Carbon Use Efficiency in High-Organic Matter Soils. mBio.

[66] Sardans J., Peñuelas J., Ogaya R. (2008). Experimental Drought Reduced Acid and Alkaline Phosphatase Activity and Increased Organic Extractable P in Soil in a Quercus Ilex Mediterranean Forest. Eur. J. Soil. Biol..

[67] Díaz-Pereira E., Marín Sanleandro P., Asencio A.D. (2019). Effects of Drought and Water Pulses on Microbial Functionality and the Role of Cyanoprokaryota in the Rhizospheres of Gypsophytes. Sci. Total Environ..

[68] Sun C., Wang R., Tang G., Cai S., Shi H., Liu F., Xie H., Zhu J., Xiong Q. (2023). Integrated 16S and Metabolomics Revealed the Mechanism of Drought Resistance and Nitrogen Uptake in Rice at the Heading Stage under Different Nitrogen Levels. Front. Plant Sci..

[69] Campdelacreu Rocabruna P., Domene X., Preece C., Fernández-Martínez M., Maspons J., Peñuelas J. (2024). Effect of Climate, Crop, and Management on Soil Phosphatase Activity in Croplands: A Global Investigation and Relationships with Crop Yield. Eur. J. Agron..

[70] Tang H., Xiao X., Li C., Shi L., Cheng K., Li W., Wen L., Xu Y., Wang K. (2021). Microbial Carbon Source Utilization in Rice Rhizosphere Soil with Different Tillage Practice in a Double Cropping Rice Field. Sci. Rep..

[71] Dong L., Bai X., Hu S., Zhang M., Wang Y., Yu X. (2024). Effects of Soil Bacterial Taxa under Different Precipitation Gradients on the Multi-Functionality of the Rhizosphere Soils under Caragana Intermedia Forests. Sustainability.

[72] Naidoo Y., Valverde A., Pierneef R.E., Cowan D.A. (2022). Differences in Precipitation Regime Shape Microbial Community Composition and Functional Potential in Namib Desert Soils. Microb. Ecol..

[73] Cordero I., Leizeaga A., Hicks L.C., Rousk J., Bardgett R.D. (2023). High Intensity Perturbations Induce an Abrupt Shift in Soil Microbial State. ISME J..

[74] Meehan C.J., Beiko R.G. (2014). A Phylogenomic View of Ecological Specialization in the Lachnospiraceae, a Family of Digestive Tract-Associated Bacteria. Genome Biol. Evol..

[75] Ormeño-Orrillo E., Martínez-Romero E. (2019). A Genomotaxonomy View of the Bradyrhizobium Genus. Front. Microbiol..

[76] Igwe A.N., Pearse I.S., Aguilar J.M., Strauss S.Y., Vannette R.L. (2024). Plant Species within Streptanthoid Complex Associate with Distinct Microbial Communities That Shift to Be More Similar under Drought. Ecol. Evol..

[77] Almonacid-Muñoz L., Herrera H., Fuentes-Ramírez A., Vargas-Gaete R., Toy-Opazo O., De Oliveira Costa P.H., Da Silva Valadares R.B. (2024). What Fire Didn’t Take Away: Plant Growth-Promoting Microorganisms in Burned Soils of Old-Growth Nothofagus Forests in Los Andes Cordillera. Plant Soil.

[78] Blacher E., Bashiardes S., Shapiro H., Rothschild D., Mor U., Dori-Bachash M., Kleimeyer C., Moresi C., Harnik Y., Zur M. (2019). Potential Roles of Gut Microbiome and Metabolites in Modulating ALS in Mice. Nature.

[79] Özdemir A., Erguven G.O., Adar E., Nuhoglu Y. (2020). Investigation on Microbial Biodeterioration of the Stone Monuments in Yildiz Technical University—Yildiz Campus—Istanbul—Turkey. Curr. Microbiol..

[80] Jurburg S.D., Natal-da-Luz T., Raimundo J., Morais P.V., Sousa J.P., Van Elsas J.D., Salles J.F. (2018). Bacterial Communities in Soil Become Sensitive to Drought under Intensive Grazing. Sci. Total Environ..

[81] Maisnam P., Jeffries T.C., Szejgis J., Bristol D., Singh B.K., Eldridge D.J., Horn S., Chieppa J., Nielsen U.N. (2023). Severe Prolonged Drought Favours Stress-Tolerant Microbes in Australian Drylands. Microb. Ecol..

[82] Khan M.R., Mohiddin F.A. (2018). Trichoderma: Its Multifarious Utility in Crop Improvement. Crop Improvement Through Microbial Biotechnology.

[83] Abdel-Azeem A.M., Abdel-Azeem A.M. (2020). Taxonomy and Biodiversity of the Genus Chaetomium in Different Habitats. Recent Developments on Genus Chaetomium.

[84] Alster C.J., Allison S.D., Johnson N.G., Glassman S.I., Treseder K.K. (2021). Phenotypic Plasticity of Fungal Traits in Response to Moisture and Temperature. ISME Commun..

[85] Lozano Y.M., Aguilar-Trigueros C.A., Roy J., Rillig M.C. (2021). Drought Induces Shifts in Soil Fungal Communities That Can Be Linked to Root Traits across 24 Plant Species. New Phytol..

[86] Li F., Chen L., Zhao Z.-H., Li Y., Yu H.-Y., Wang Y., Zhang J.-B., Han Y.-L. (2023). The Changes of Chemical Molecular Components in Soil Organic Matter Are Associated with Fungus Mortierella Capitata K. Soil. Tillage Res..

[87] Dikilitas M., Karakas S., Hashem A., Abd Allah E.F., Ahmad P. (2016). Oxidative Stress and Plant Responses to Pathogens under Drought Conditions. Water Stress and Crop Plants.

[88] Niaz K., Rauf M., Arif M., Hamayun M., Gul H., Hashem A., Abd_Allah E.F., Wu Q.-S. (2024). Drought-Tolerant Fungal Microbes, Aspergillus Oryzae and Aspergillus Fumigatus, Elevate Physiohormonal and Antioxidant Responses of Maize under Drought Stress. Front. Microbiol..

[89] Hu J., Miller G., Shi W. (2023). Abundance, Diversity, and Composition of Root-Associated Microbial Communities Varied with Tall Fescue Cultivars under Water Deficit. Front. Microbiol..

[90] Delgado-Baquerizo M., Oliverio A.M., Brewer T.E., Benavent-González A., Eldridge D.J., Bardgett R.D., Maestre F.T., Singh B.K., Fierer N. (2018). A Global Atlas of the Dominant Bacteria Found in Soil. Science.

[91] Li X., He C., He X., Su F., Hou L., Ren Y., Hou Y. (2019). Dark Septate Endophytes Improve the Growth of Host and Non-Host Plants under Drought Stress through Altered Root Development. Plant Soil.

[92] Buzzini P., Turchetti B., Yurkov A. (2018). Extremophilic Yeasts: The Toughest Yeasts Around?. Yeast.

[93] Glushakova A.M., Lysak L.V., Kachalkin A.V., Ivanova A.E., Umarova A.B., Abramyan I.A., Ezhelev Z.S., Maksimova I.A. (2021). Transformation of Microbial Complexes in Components of Soil Constructions of Different Origin (Soil, Peat, Sand) during Freezing-Thawing Processes. Microbiology.

[94] Marçais B., Kosawang C., Laubray S., Kjær E., Kirisits T. (2022). Ash Dieback. Forest Microbiology.

[95] Lilleskov E.A., Kuyper T.W., Bidartondo M.I., Hobbie E.A. (2019). Atmospheric Nitrogen Deposition Impacts on the Structure and Function of Forest Mycorrhizal Communities: A Review. Environ. Pollut..

[96] Treseder K.K., Berlemont R., Allison S.D., Martiny A.C. (2018). Drought Increases the Frequencies of Fungal Functional Genes Related to Carbon and Nitrogen Acquisition. PLoS ONE.

