# Supplementary material for: Unraveling the Effect of Soil Moisture on Microbial Diversity and Enzymatic Activity in Agricultural Soils
Source: Microorganisms. 2025 May 28;13(6):1245. doi: 10.3390/microorganisms13061245 (PMC12195373; doi:10.3390/microorganisms13061245)
Supplement: Supplementary file 1 [file microorganisms-13-01245-s001.zip › microorganisms-3640953-supplementary.pdf]

Table S1. Media composition (g/L)

Plate count agar (PCA agar, Biomaxima)	Rose Bengal agar (Biomaxima)	Actinomycete isolation agar (Becton Dickinson)
Enzymatic hydrolysate of casein - 5.0	Bacteriological peptone - 5.0	Sodium caseinate - 2.0
Yeast extract - 2.5	Rose Bengal - 0.05	Asparagine - 0.1
Glucose - 1.0	K_2PO_4 - 1.0	Sodium propionate - 4.0
Agar - 15.0	$MgSO_4$ - 0.5	K_2PO_4 - 0.5
	Glucose - 10.0	$MgSO_4$ - 0.1
	Agar - 15.0	$FeSO_4$ - 0.001
		Agar - 15.0

Table S2. Pearson correlation matrix with respective r values between soil moisture and physicochemical at zero sampling day (T0) and 8th week (T8) of induced drought conditions (G; Gniewkowo, L; Lulkowo, N; Nieszawa, S; Suchatówka, C; organic carbon, $CaCO_3$: calcium carbonate, N; total nitrogen, NO_3^-; nitrate, NH_4^+; ammonium, P; total phosphorus, P_2O_5; available phosphorus, NA; Not applicable).

	**G0**	**G8**	**L0**	**L8**	**N0**	**N8**	**S0**	**S8**
**C**	0.58	0.19	0.95	0.58	0.55	0.12	0.94	0.73
**N**	0.99	0.62	0.88	0.73	0.70	0.87	0.26	0.80
**P**	0.48	0.68	0.95	0.80	0.72	0.87	0.74	0.83
**P_2O_5**	0.52	0.81	0.95	0.58	0.81	0.76	0.94	0.73
**NO_3^-**	0.18	0.99	0.82	0.80	0.52	0.80	0.98	0.31
**NH_4^+**	0.96	0.19	0.91	0.73	0.77	0.76	0.77	0.86
**pH**	0.67	0.76	0.98	0.29	0.64	0.95	0.94	0.90
**$CaCO_3$**	0.41	0.98	NA	NA	0.48	0.87	0.94	0.39

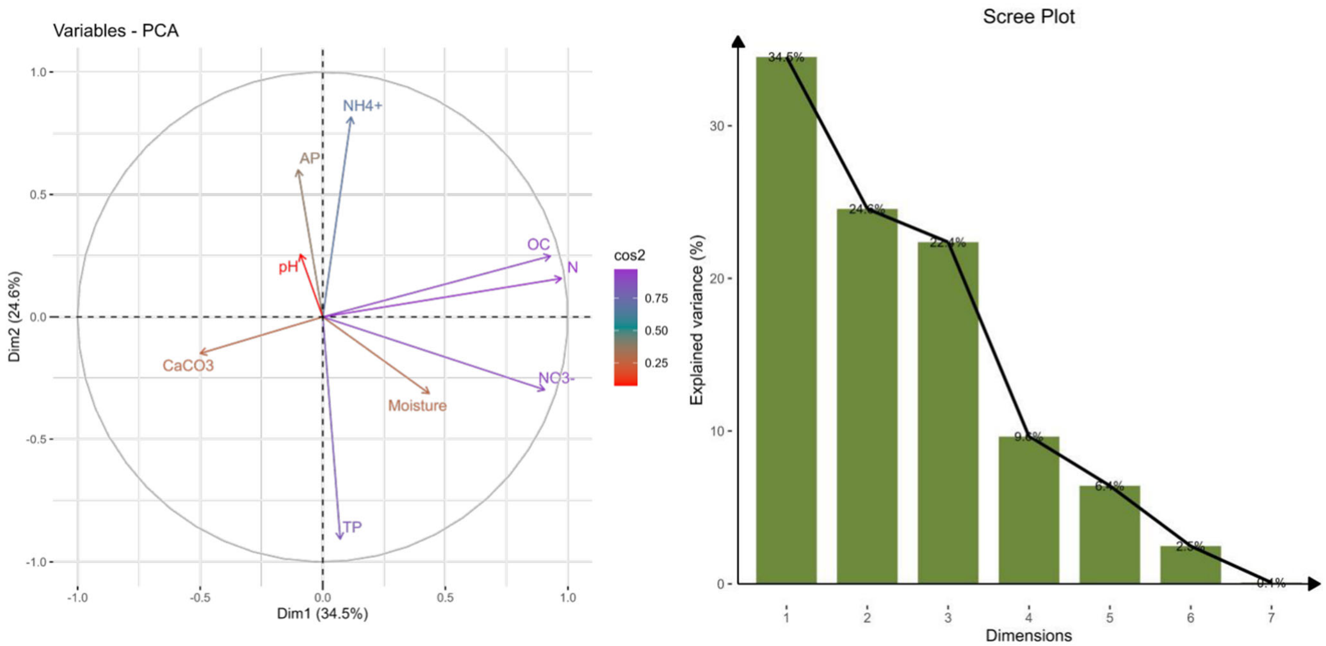

A B

Figure S1. Principal component analysis (PCA) of (A) the studied correlations between soil moisture content and physicochemical parameters at T0 and T8, (B) the respective contribution of each component to their total variability. G, Gniewkowo; L, Lulkowo; N, Wielka Nieszawa; S, Suchatówka; 0, week 0; 8, week 8.

Table S3. Enumeration of bacteria, fungi and actinomycetes in four agricultural soils (CFU g−1 dry soil) (G; Gniewkowo, L; Lulkowo, N; Nieszawa, S; Suchatówka).

	**Microorganisms**											
	**Bacteria x 10^4**				**Actinomycetes x 10^4**				**Fungi x 10^2**			
**Weeks**	**G**	**L**	**N**	**S**	**G**	**L**	**N**	**S**	**G**	**L**	**N**	**S**
**0**	946.7	560.0	663.3	400.0	27.1	11.2	19.1	19.5	121.0	101.7	55.7	54.0
**1**	1,160.0	530.0	950.0	473.3	8.0	7.0	27.9	17.4	86.3	82.7	72.0	52.0
**2**	560.0	370.0	660.0	470.0	14.5	6.6	9.5	11.2	66.3	55.3	68.7	30.7
**4**	292.7	167.3	650.0	284.7	13.9	7.4	11.1	10.3	67.0	54.0	58.3	56.3
**8**	284.0	154.7	201.7	187.7	9.7	8.5	18.8	10.2	84.0	104.7	52.0	37.7

Table S4. Pearson correlation matrix with respective r values between soil moisture and biological parameters at 0, 1, 2, 4, and 8 weeks of induced drought conditions (G; Gniewkowo, L; Lulkowo, N; Nieszawa, S; Suchatówka, PH-AC, Acid phosphatase; PH-AL, Alkaline phosphatase; DH, Dehydrogenase; UR, Urease; 0, week 0; 8, week 8).

	G0	G8	L0	L8	N0	N8	S0	S8
**DH**	0.42	0.77	0.98	0.85	0.02	0.43	0.93	0.06
**PH-AC**	0.96	0.02	0.21	0.69	0.02	0.63	0.02	0.90
**PH-AL**	0.27	0.94	0.99	0.48	1.00	0.98	0.48	0.24
**UR**	0.09	0.76	0.98	0.96	0.77	NA*	0.52	0.59
**Bacteria**	0.58	0.98	0.67	0.97	0.94	0.50	0.48	0.99
**Actinomycetes**	0.97	0.33	0.01	0.16	0.92	0.81	0.97	0.08
**Fungi**	0.20	0.77	0.15	0.83	0.66	0.94	0.97	0.48

*NA=not applicable.

Table S5. Pearson correlation matrix with respective r values between microbial abundance and enzymatic activities at T0 and T8 weeks under induced drought conditions. G; Gniewkowo, L; Lulkowo, N; Nieszawa, S; Suchatówka, PH-AC, Acid phosphatase; PH-AL, Alkaline phosphatase; DH, Dehydrogenase; UR, Urease.

	G0	G8	L0	L8	N0	N8	S0	S8
**A. Bacteria**								
**PH-AC**	0.33	0.20	0.87	0.84	0.33	0.36	0.87	0.84
**PH-AL**	0.94	0.85	0.54	0.67	0.94	0.67	1.00	0.12
**DH**	0.50	0.87	0.50	0.95	0.33	1.00	0.11	0.18
**UR**	-0.86	0.87	0.50	0.87	0.50	0.87	0.50	0.68
**B. Actinomycetes**								
**PH-AC**	-0.87	0.95	0.98	0.61	0.41	0.05	0.23	0.51
**PH-AL**	-0.49	0.02	0.16	0.79	0.92	0.91	0.69	0.99
**DH**	0.19	0.86	0.20	0.37	0.41	0.88	0.80	0.99
**UR**	0.33	0.87	0.20	0.44	0.96	0.59	0.28	0.76
**C. Fungi**								
**PH-AC**	-0.46	0.65	1.00	0.97	0.76	0.34	0.23	0.81
**PH-AL**	0.89	0.50	0.02	0.88	0.67	0.99	0.69	0.97
**DH**	0.97	1.00	0.06	1.00	0.76	0.70	0.80	0.85
**UR**	-0.96	1.00	0.06	0.64	0.99	0.33	0.29	0.43

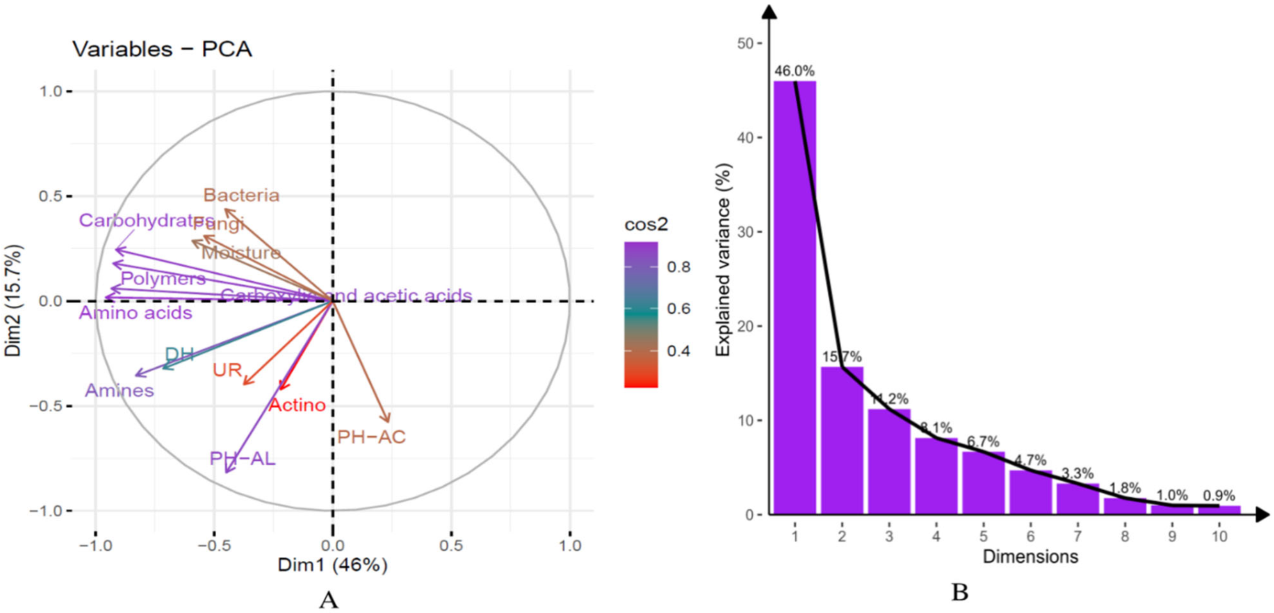

Figure S2. Principal component analysis (PCA) indicating correlations between soil moisture content and biological parameters in four soil samples at investigated time intervals. A) the studied correlations between soil moisture content and biological parameters at T0 and T8, (B) the respective contribution of each component to their total variability. G, Gniewkowo; L, Lulkowo; N, Wielka Nieszawa; S, Suchatówka; Actino, Actinomycetes; PH-AC, Acid phosphatase; PH-AL, Alkaline phosphatase; DH, Dehydrogenase; UR, Urease; 0, week 0; 1, week 1; 2, week 2; 4, week 4; 8, week 8.

Table S6. Pearson correlation matrix with respective r values between soil moisture and major carbon sources (carbohydrates (CH), carboxylic and acetic acids (CA), amino acids (AA), amines (AM), and polymers (PL)) at T0 and T8 week of induced drought conditions. G; Gniewkowo, L; Lulkowo, N; Nieszawa, S; Suchatówka, 0; sampling day, 8; 8th week.

	**G0**	**G8**	**L0**	**L8**	**N0**	**N8**	**S0**	**S8**
**CA**	0.76	0.60	0.69	0.33	0.91	0.97	0.66	1.00
**CH**	0.34	0.72	0.96	0.61	0.27	0.64	0.90	0.30
**PL**	0.30	0.66	0.69	0.67	0.31	0.98	0.98	0.11
**AA**	0.40	0.57	0.38	0.88	0.97	0.46	0.11	0.73
**AM**	0.98	0.63	0.79	0.78	0.36	0.16	0.75	0.57

Table S7. Pearson correlation matrix with respective r values between soil physicochemical and biological factors at zero sampling day (T0) and 8th week (T8) of induced drought conditions (G; Gniewkowo, L; Lulkowo, N; Nieszawa, S; Suchatówka, C; organic carbon, $CaCO_3$; calcium carbonate, N; total nitrogen, NO_3^-; nitrate, NH_4^+; ammonium, P; total phosphorus, P_2O_5; available phosphorus, NA; Not applicable; DH; Dehydrogenase, PH-AC; Acid phosphatase, PH-AL; Alkaline phosphatase, UR; Urease, CA; carboxylic and acetic acids, CH; carbohydrates, PL; polymers, AA; amino acids, AM; amines, and PL; polymers).

	DH		PH-AC		PH-AL		UR		Bacteria		Actinomycetes		Fungi		CA		CH		PL		AA		AM	
	**G0**	**G8**	**G0**	**G8**	**G0**	**G8**	**G0**	**G8**	**G0**	**G8**	**G0**	**G8**	**G0**	**G8**	**G0**	**G8**	**G0**	**G8**	**G0**	**G8**	**G0**	**G8**	**G0**	**G8**
**Soil moisture**	0.42	0.77	0.96	0.02	0.27	0.94	0.09	0.76	0.58	0.98	0.97	0.33	0.2	0.77	0.76	0.97	0.34	0.48	0.3	0.96	0.4	0.21	0.98	0.17
**C**	0.5	0.49	0.33	0.98	0.94	0.52	0.87	0.5	1	0	0.75	0.87	0.69	0.49	0.09	0.4	0.57	0.77	0.61	0.46	0.52	1	0.73	1
**N**	0.29	0.98	0.91	0.79	0.4	0.31	0.23	0.98	0.68	0.76	0.99	0.94	0.06	0.98	0.67	0.43	0.21	0.99	0.17	0.37	0.27	0.63	1	0.67
**P**	0.6	0.99	0.22	0.75	0.97	0.38	0.92	0.99	0.99	0.8	0.67	0.92	0.77	0.99	0.2	0.5	0.66	0.97	0.69	0.44	0.61	0.57	0.65	0.61
**P_2O_5**	0.56	1	0.26	0.6	0.96	0.55	0.9	1	1	0.9	0.71	0.82	0.74	1	0.16	0.66	0.63	0.9	0.66	0.61	0.57	0.4	0.69	0.44
**NO_3^-**	0.82	0.69	0.1	0.09	1	0.97	1	0.68	0.91	0.96	0.41	0.23	0.93	0.69	0.5	0.99	0.86	0.38	0.88	0.98	0.83	0.32	0.38	0.28
**NH_4^+**	0.16	0.49	0.85	0.98	0.52	0.52	0.36	0.5	0.78	0	1	0.87	0.08	0.49	0.56	0.4	0.07	0.77	0.03	0.46	0.14	1	1	1
**pH**	0.4	0.17	0.43	0.63	0.9	0.94	0.8	0.16	0.99	0.63	0.82	0.36	0.6	0.17	0.03	0.89	0.48	0.2	0.51	0.91	0.42	0.8	0.81	0.77
**$CaCO_3$**	0.65	0.87	0.14	0.2	0.99	0.85	0.94	0.87	0.98	1	0.62	0.5	0.81	0.87	0.28	0.92	0.72	0.64	0.75	0.89	0.67	0.03	0.59	0.01

	**L0**	**L8**	**L0**	**L8**	**L0**	**L8**	**L0**	**L8**	**L0**	**L8**	**L0**	**L8**	**L0**	**L8**	**L0**	**L8**	**L0**	**L8**	**L0**	**L8**	**L0**	**L8**	**L0**	**L8**
**Soil moisture**	0.98	0.85	0.21	0.69	0.99	0.48	0.98	0.96	0.67	0.97	0.01	0.16	0.15	0.83	0.69	0.33	0.95	0.61	0.69	0.67	0.38	0.88	0.79	0.78
**C**	0.87	0.92	0.5	0.99	0.89	0.99	0.87	0.33	0.87	0.76	0.32	0.71	0.45	0.94	0.43	0.58	0.82	1	0.43	0.99	0.07	0.9	0.56	0.06
**N**	0.76	0.98	0.65	1	0.78	0.95	0.76	0.5	0.94	0.87	0.49	0.56	0.61	0.99	0.25	0.41	0.69	0.99	0.25	1	0.12	0.97	0.39	0.13
**P**	0.87	1	0.5	0.99	0.89	0.91	0.87	0.6	0.87	0.92	0.32	0.46	0.45	1	0.43	0.3	0.82	0.96	0.43	0.98	0.07	0.99	0.56	0.24
**P_2O_5**	0.87	0.92	0.5	0.99	0.89	0.99	0.87	0.33	0.87	0.76	0.32	0.71	0.45	0.94	0.43	0.58	0.82	1	0.43	0.99	0.07	0.9	0.56	0.06
**NO_3^-**	0.92	1	0.4	0.99	0.9	0.91	0.92	0.6	0.11	0.92	0.57	0.46	0.45	1	0.98	0.3	0.95	0.96	0.98	0.98	0.84	0.99	1	0.24
**NH_4^+**	0.8	0.98	0.6	1	0.83	0.95	0.8	0.5	0.92	0.87	0.43	0.56	0.55	0.99	0.33	0.41	0.74	0.99	0.32	1	0.04	0.97	0.46	0.13
**pH**	1	0.74	0	0.89	1	0.98	1	0	0.5	0.5	0.2	0.9	0.06	0.77	0.83	0.81	1	0.94	0.82	0.9	0.56	0.71	0.9	0.38
**$CaCO_3$**	0	0	0	0	0	0	0	0	0	0	0	0	0	0	0	0	0	0	0	0	0	0	0	0

	**N0**	**N8**	**N0**	**N8**	**N0**	**N8**	**N0**	**N8**	**N0**	**N8**	**N0**	**N8**	**N0**	**N8**	**N0**	**N8**	**N0**	**N8**	**N0**	**N8**	**N0**	**N8**	**N0**	**N8**
**Soil moisture**	0.02	0.42	0.02	0.63	1	0.98	0.77	0	0.94	0.5	0.92	0.81	0.66	0.94	0.91	0.97	0.27	0.64	0.31	0.98	0.97	0.46	0.36	0.16
**C**	0.83	0.95	0.83	0.7	0.54	0.32	0.11	0.99	0.8	0.92	0.17	0.68	0.26	0.44	0.14	0.35	0.65	0.69	0.62	0.1	0.31	0.83	0.59	0.96
**N**	0.7	0.82	0.7	0.16	0.7	0.95	0.08	0.5	0.9	0.87	0.36	0.99	0.07	0.98	0.33	0.96	0.49	0.18	0.46	0.74	0.49	0.05	0.42	0.35
**P**	0.68	0.08	0.68	0.93	0.72	0.74	0.11	0.5	0.92	0	0.39	0.41	0.04	0.65	0.36	0.72	0.47	0.94	0.43	0.95	0.52	0.84	0.39	0.63

**P_2O_5**	0.58	0.27	0.58	0.98	0.8	0.61	0.24	0.65	0.96	0.19	0.51	0.23	0.09	0.49	0.48	0.58	0.35	0.99	0.31	0.88	0.63	0.93	0.27	0.77
**NO_3^-**	0.87	0.88	0.87	0.04	0.52	0.91	0.94	0.6	0.19	0.92	0.81	1	0.98	0.96	0.83	0.92	0.97	0.06	0.97	0.66	0.72	0.16	0.98	0.46
**NH_4^+**	0.62	0.91	0.62	0.03	0.77	0.87	0.19	0.65	0.94	0.94	0.46	1	0.04	0.93	0.43	0.89	0.4	0.01	0.36	0.6	0.58	0.24	0.32	0.52
**pH**	0.76	0.7	0.76	0.34	0.63	0.99	0	0.33	0.87	0.76	0.29	0.96	0.15	1	0.25	1	0.56	0.36	0.53	0.86	0.42	0.14	0.49	0.17
**$CaCO_3$**	0.87	0.82	0.87	0.16	0.48	0.95	0.19	0.5	0.76	0.87	0.1	0.99	0.34	0.98	0.06	0.96	0.71	0.18	0.68	0.74	0.24	0.05	0.65	0.35

	**S0**	**S8**	**S0**	**S8**	**S0**	**S8**	**S0**	**S8**	**S0**	**S8**	**S0**	**S8**	**S0**	**S8**	**S0**	**S8**	**S0**	**S8**	**S0**	**S8**	**S0**	**S8**	**S0**	**S8**
**Soil moisture**	0.93	0.06	0.02	0.9	0.48	0.24	0.52	0.59	0.48	0.99	0.97	0.08	0.97	0.48	0.66	1	0.9	0.3	0.98	0.11	0.11	0.73	0.75	0.57
**C**	0.74	0.73	0.33	0.35	0.76	0.5	0.19	0.98	0.76	0.8	1	0.63	0.99	0.26	0.36	0.73	0.69	0.87	0.99	0.61	0.24	1	0.47	0.15
**N**	0.13	0.65	0.96	0.46	0.97	0.39	0.69	0.96	0.97	0.87	0.5	0.53	0.49	0.14	0.56	0.8	0.19	0.81	0.44	0.51	0.93	0.99	0.45	0.04
**P**	0.43	0.51	0.65	0.99	0.94	0.74	0.19	0.04	0.94	0.76	0.89	0.62	0.89	0.89	0.01	0.83	0.38	0.29	0.86	0.64	0.58	0.22	0.11	0.93
**P_2O_5**	0.74	0.73	0.33	0.35	0.76	0.5	0.19	0.98	0.76	0.8	1	0.63	0.99	0.26	0.36	0.73	0.69	0.87	0.99	0.61	0.24	1	0.47	0.15
**NO_3^-**	0.83	0.93	0.19	0.69	0.65	1	0.33	0.59	0.65	0.19	1	0.97	1	0.98	0.49	0.3	0.79	0.82	1	0.98	0.1	0.43	0.59	0.96
**NH_4^+**	0.95	0.55	0.65	0.56	0.19	0.29	0.94	0.92	0.19	0.92	0.58	0.43	0.58	0.03	0.99	0.87	0.97	0.74	0.63	0.41	0.72	0.97	1	0.08
**pH**	0.74	0.49	0.33	0.62	0.76	0.21	0.19	0.88	0.76	0.94	1	0.36	0.99	0.04	0.36	0.9	0.69	0.69	0.99	0.34	0.24	0.95	0.47	0.15
**$CaCO_3$**	0.74	0.94	0.33	0.05	0.76	0.8	0.19	0.97	0.76	0.5	1	0.89	0.99	0.62	0.36	0.4	0.69	0.99	0.99	0.87	0.24	0.92	0.47	0.53

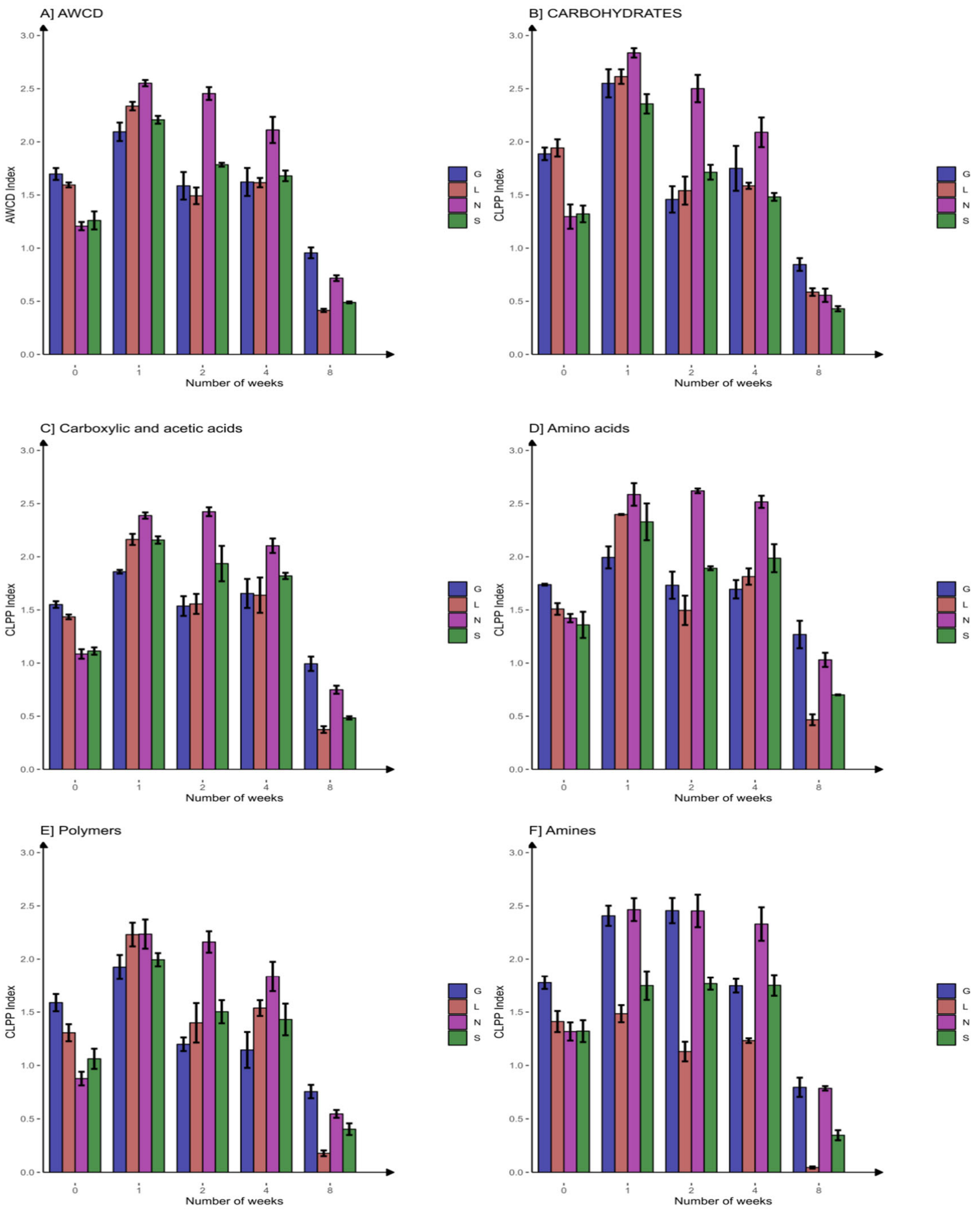

Figure S3. Absorbance values of Biolog-Ecoplates in four types of agricultural soil samples with carbon substrate utilization efficiency. (A) Average rate of the average well color development (AWCD) over the incubation time (ΔAWCD/weeks); (B) Carbohydrates metabolism; (C) Carboxylic and acetic acids metabolism; (D) Amino acids metabolism; (E) Amine's metabolism; (F) Polymers metabolism. G, Gniewkowo; L, Lulkowo; N, Wielka Nieszawa; S, Suchatówka.

Result

S3.2. Microbial composition and diversity

Crenarchaeota abundance increased in G (0.14% → 1.04%), S (2.09% → 4.23%), and N sites (1.31% → 0.33%) (Figure 8). Gemmatimonadota showed minimal changes in G (1.86% → 1.83%) and slight increases in L (2.80% → 2.84%) and N sites (0.67% → 2.91%). Nitrospirota shows drastic increases in L (0.62% → 3.47%) and S site (2.09% → 4.23%) (Table 5). *Pseudarthrobacter* declines significantly in G (2.49% → 1.09%) and L site (0.16% → 0.36%). *Nitrospira* increases across all sites (G: 0.26% → 0.72%, L: 0.08% → 0.54%, N: 0.46% → 0.62%; S: 0.31% → 1.10%) (Figure 9). *Pseudolabrys* also demonstrates large variations in abundance, with a peak at L0 (2.6%) and near-zero abundance at N0. *Hassallia* increases in G (0.29% → 0.38%) and S site (0.45% → 0.95%). *RB41* increases in all soil types, especially in S site (0.64% → 1.17%). *Pseudolabrys* abundance decreased drastically in L site from 2.62% → 0.08%). *Nitrospira* and *Parabacteroides* on the other hand, are relatively stable across all conditions, indicating this genus may be more resilient to drought stress (Figure 9; Table 4).

Mortierellomycota shows an increase in abundance in G (1.59%), L (9.41%), and N (1.67%), but decreased in S (4.42%) site. Chytridiomycota shows a notable increase in G (0.63%), L (4.15%) and N (2.79%) (Figure 11; Table 6). *Plectosphaerella* showed a decline in G (21.338%) and S (0.085%) but increased in L (0.592%) and N (0.885%) sites. *Ramophialophora* showed an increase in S (7.21%). *Trichoderma* increased in L (6.32%) site (Figure 12; Table 6).

Discussion

S4.5. Impact of drought stress on total microbial communities (16S and ITS)

In oligotrophic and drought-stressed conditions, Crenarchaeota—in particular, ammonia-oxidizing archaea (AOA)—are renowned for their adaptability. As organic nitrogen inputs are reduced by drought, their increase in most sites indicates an increased reliance on ammonia oxidation for nitrogen cycling [1]. According to [2], Gemmatimonadota are renowned for their drought adaptation strategies, which include fast osmotic adjustment and dormancy. Their relative stability indicates that they are already acclimated to arid environments and sustain population sizes despite variations in soil moisture. The rise in N soil indicates that this phylum might be involved in the cycling of carbon and phosphorus, especially in drought-affected situations with minimum microbial competition [2]. The process of nitrification, or the transformation of nitrite into nitrate, depends on Nitrospirota, which contains Nitrospira. Their increase in abundance indicates a change toward microbially driven nitrogen cycling, most likely because of less competition from nitrogen-fixing bacteria, which usually decrease during dry conditions [3]. Nitrification is still active in well-aerated conditions even under drought stress, as evidenced by the largest increase in S and L sites [4].

Parabacteroides is a gut-associated species that also grows well in soil and is involved in the breakdown of polysaccharides. Its decrease suggests a decrease in drought-stressed microbially mediated glucose breakdown [5]. *Pseudarthrobacter* is renowned for its ability to degrade hydrocarbons and withstand drought. Its capacity to remain stable in the S site indicates that it contributes to microbial survival in environments with limited water [6]. *Pseudolabrys* is an oligotrophic bacterium, capable of surviving in nutrient-poor conditions. Its decline in L site suggests that drought may reduce its ecological niche by altering soil structure and nutrient flow [7]. Stress tolerance and the breakdown of soil organic materials are associated with RB41 (Acidobacteria). Its increase in S site indicates that it has adapted to severe drought circumstances, maybe as a result of its capacity to endure low soil moisture levels [7].

The significant increases in G (0.63%), L (4.15%), and N (2.79%) observed in Chytridiomycota may also be related to its adaptability to changing soil moisture levels. Chytridiomycetes may benefit from temporary soil moisture availability even during drought conditions, as they flourish in aquatic or water-retaining microenvironments [8,9]. Their capacity to break down complex sugars found in

plant residues could also give them a competitive edge when microbial interactions are altered by drought [8,9]. The bacterial Shannon index indicates that bacterial communities are drought-tolerant, which may be because of the changes in dominant species or the presence of drought-adapted taxa [10,11]. This increase was most pronounced in the site L soil (6.33 → 7.04), suggesting the ability of soil to retain soil moisture may help bacterial communities to survive dryness. However, the bacterial Simpson Index suggests that there was no significant loss of evenness or domination by drought-tolerant taxa, even though there may have been some changes in the community composition [12,13]. The bacterial population in the soil types under investigation show modest changes in diversity and a strong tolerance to drought stress. According to these changes in fungal phylum abundance, fungal communities are significantly shaped by site-specific variables and drought. While Mortierellomycota and Chytridiomycota have increased, suggesting possible resilience and adaptation to dry conditions, Ascomycota and Basidiomycota have declined across most sites, highlighting their vulnerability to soil moisture stress. Since changes in fungal populations can affect nutrient cycling, organic matter decomposition, and plant-microbe interactions, it is imperative to comprehend these responses as they ultimately impact the stability of soil ecosystems under drought stress brought on by climate change.

The soilborne plant pathogen *Plectosphaerella* grows best in conditions that are high in organic matter. According to [14], the drop observed in humus-rich G soil implies that drought decreased the availability of plant material, which in turn affected its survival capacity. According to [15], the modest rise in clay soils (N and L) suggests that compact, moisture-retaining soils may offer some protection against drought stress.

It is yet unknown what function the genus *Ramophialophora* plays in soil [16]. *Trichoderma* grew in L (6.32%), confirming its role as a drought-resistant, plant-associated fungus. Trichoderma species improve plant stress tolerance, decompose organic matter, and act as biocontrol agents [17]. The increase in clay-sandy soils (L) suggests a favorable niche where *Trichoderma* can maintain root associations and exploit plant-stress responses [18]. According to the fungal Shannon index, community composition may have changed because of drought stress favoring drought-tolerant fungi. The greatest increase in G soil (1.16 units) suggests that fungi that are acclimated to breaking down organic materials may have multiplied under stress [19]. The decline in S site indicates that fungus richness in well-draining, low-nutrient sandy soil was significantly impacted negatively by drought. Overall diversity may have decreased because of fungal die-off or the dominance of a few particularly drought-resistant species due to reduced ability of sandy soil to retain moisture [20]. The Simpson index's indication of a balanced fungal community may result from the decline of dominant species and the rise of less prevalent, stress-tolerant fungi [21,22].

References

1. Yrjälä, K.; Lopez-Echartea, E. Archaea as Components of Forest Microbiome. In *Forest Microbiology*; Elsevier, 2021; pp. 357–370 ISBN 978-0-12-822542-4.
2. Yuan, A.; Kumar, S.D.; Wang, H.; Wang, S.; Impa, S.; Wang, H.; Guo, J.; Wang, Y.; Yang, Q.; Liu, X.J.A.; et al. Dynamic Interplay among Soil Nutrients, Rhizosphere Metabolites, and Microbes Shape Drought and Heat Stress Responses in Summer Maize. *Soil Biology and Biochemistry* **2024**, *191*, 109357, doi:10.1016/j.soilbio.2024.109357.
3. Huang, Y.; Li, L.; Li, G.; Peng, X.; Ou, G.; Zhou, J.; Qin, F.; Yang, W.; Wei, M.; Shen, Z. Fenlong Tillage Accelerates the Enrichment of the Host-specific Rhizosphere Bacterial Community of Cassava. *Soil Use and Management* **2025**, *41*, e70025, doi:10.1111/sum.70025.
4. Gu, Z.; Hu, C.; Gan, Y.; Zhou, J.; Tian, G.; Gao, L. Role of Microbes in Alleviating Crop Drought Stress: A Review. *Plants* **2024**, *13*, 384, doi:10.3390/plants13030384.
5. Wexler, A.G.; Goodman, A.L. An Insider's Perspective: Bacteroides as a Window into the Microbiome. *Nat Microbiol* **2017**, *2*, 17026, doi:10.1038/nmicrobiol.2017.26.

6. Igwe, A.N.; Pearse, I.S.; Aguilar, J.M.; Strauss, S.Y.; Vannette, R.L. Plant Species within Streptanthoid Complex Associate with Distinct Microbial Communities That Shift to Be More Similar under Drought. *Ecology and Evolution* **2024**, *14*, e11174, doi:10.1002/ece3.11174.
7. Liu, S.; Liu, J.; She, J.; Xie, Z.; Zhou, L.; Dai, Q.; Zhang, X.; Wan, Y.; Yin, M.; Dong, X.; et al. Microbial Features with Uranium Pollution in Artificial Reservoir Sediments at Different Depths under Drought Stress. *Science of The Total Environment* **2024**, *919*, 170694, doi:10.1016/j.scitotenv.2024.170694.
8. Hanrahan-Tan, D.G.; Lilje, O.; Henderson, L. Chytrids in Soil Environments: Unique Adaptations and Distributions. *Encyclopedia* **2023**, *3*, 642–664, doi:10.3390/encyclopedia3020046.
9. Vikram, S.; Ramond, J.-B.; Ortiz, M.; Maggs-Kölling, G.; Pelser, K.; Cowan, D.A. Soil Fungal Diversity and Assembly along a Xeric Stress Gradient in the Central Namib Desert. *Fungal Biology* **2023**, *127*, 997–1003, doi:10.1016/j.funbio.2023.03.001.
10. Naylor, D.; Coleman-Derr, D. Drought Stress and Root-Associated Bacterial Communities. *Front. Plant Sci.* **2018**, *8*, 2223, doi:10.3389/fpls.2017.02223.
11. Naylor, D.; DeGraaf, S.; Purdom, E.; Coleman-Derr, D. Drought and Host Selection Influence Bacterial Community Dynamics in the Grass Root Microbiome. *The ISME Journal* **2017**, *11*, 2691–2704, doi:10.1038/ismej.2017.118.
12. Barnard, R.L.; Osborne, C.A.; Firestone, M.K. Changing Precipitation Pattern Alters Soil Microbial Community Response to Wet-up under a Mediterranean-Type Climate. *The ISME Journal* **2015**, *9*, 946–957, doi:10.1038/ismej.2014.192.
13. Barnard, R.L.; Blazewicz, S.J.; Firestone, M.K. Rewetting of Soil: Revisiting the Origin of Soil CO2 Emissions. *Soil Biology and Biochemistry* **2020**, *147*, 107819, doi:10.1016/j.soilbio.2020.107819.
14. Girardi, N.; Sosa, A.L.; Loyola García, J.; Pellegrino, M.; Passone, M.A. Ecophysiological Characteristics of the Nematophagous Fungus, Plectosphaerella Plurivora, with Biocontrol Potential on Nacobbus Aberrans s.l. in Tomato. *Eur J Plant Pathol* **2023**, *167*, 867–881, doi:10.1007/s10658-023-02739-3.
15. CACCIOLA, S.O.; GULLINO, M.L. Emerging and Re-Emerging Fungus and Oomycete Soil-Borne Plant Diseases in Italy. *Phytopathologia Mediterranea* **2019**, *58*, 451–472, doi:10.14601/Phyto-10756.
16. Li, X.; Lu, Q.; Li, D.; Wang, D.; Ren, X.; Yan, J.; Ahmed, T.; Li, B. Effects of Two Kinds of Commercial Organic Fertilizers on Growth and Rhizosphere Soil Properties of Corn on New Reclamation Land. *Plants* **2022**, *11*, 2553, doi:10.3390/plants11192553.
17. Reza Boorboori, M.; Zhang, H. The Mechanisms of Trichoderma Species to Reduce Drought and Salinity Stress in Plants. *Phyton* **2023**, *92*, 2261–2281, doi:10.32604/phyton.2023.029486.
18. Cabral-Miramontes, J.P.; Olmedo-Monfil, V.; Lara-Banda, M.; Zúñiga-Romo, E.R.; Aréchiga-Carvajal, E.T. Promotion of Plant Growth in Arid Zones by Selected Trichoderma Spp. Strains with Adaptation Plasticity to Alkaline pH. *Biology* **2022**, *11*, 1206, doi:10.3390/biology11081206.
19. Glassman, S.I.; Weihe, C.; Li, J.; Albright, M.B.N.; Looby, C.I.; Martiny, A.C.; Treseder, K.K.; Allison, S.D.; Martiny, J.B.H. Decomposition Responses to Climate Depend on Microbial Community Composition. *Proc. Natl. Acad. Sci. U.S.A.* **2018**, *115*, 11994–11999, doi:10.1073/pnas.1811269115.
20. Bouskill, N.J.; Wood, T.E.; Baran, R.; Ye, Z.; Bowen, B.P.; Lim, H.; Zhou, J.; Nostrand, J.D.V.; Nico, P.; Northen, T.R.; et al. Belowground Response to Drought in a Tropical Forest Soil. I. Changes in Microbial Functional Potential and Metabolism. *Front. Microbiol.* **2016**, *7*, doi:10.3389/fmicb.2016.00525.
21. Lennon, J.T.; Jones, S.E. Microbial Seed Banks: The Ecological and Evolutionary Implications of Dormancy. *Nat Rev Microbiol* **2011**, *9*, 119–130, doi:10.1038/nrmicro2504.
22. Azarbad, H.; Constant, P.; Giard-Laliberté, C.; Bainard, L.D.; Yergeau, E. Water Stress History and Wheat Genotype Modulate Rhizosphere Microbial Response to Drought. *Soil Biology and Biochemistry* **2018**, *126*, 228–236, doi:10.1016/j.soilbio.2018.08.017.
